# Embedding a ribonuclease in the spore crust couples gene expression to spore development in *Bacillus subtilis*

**DOI:** 10.1093/nar/gkae1301

**Published:** 2025-01-16

**Authors:** Alexandre D’Halluin, Laetitia Gilet, Armand Lablaine, Olivier Pellegrini, Mónica Serrano, Anastasia Tolcan, Magali Ventroux, Sylvain Durand, Marion Hamon, Adriano O Henriques, Rut Carballido-López, Ciarán Condon

**Affiliations:** EGM CNRS, Université Paris-Cité,Institut de Biologie Physico-Chimique, 13 rue Pierre et Marie Curie, 75005 Paris, France; EGM CNRS, Université Paris-Cité,Institut de Biologie Physico-Chimique, 13 rue Pierre et Marie Curie, 75005 Paris, France; Université Paris-Saclay, INRAE, AgroParisTech, Micalis Institute, 78350 Jouy-en-Josas, France; EGM CNRS, Université Paris-Cité,Institut de Biologie Physico-Chimique, 13 rue Pierre et Marie Curie, 75005 Paris, France; Instituto de Tecnologia Química e Biológica António Xavier, Universidade Nova de Lisboa, 2780-157Oeiras, Portugal; EGM CNRS, Université Paris-Cité,Institut de Biologie Physico-Chimique, 13 rue Pierre et Marie Curie, 75005 Paris, France; Université Paris-Saclay, INRAE, AgroParisTech, Micalis Institute, 78350 Jouy-en-Josas, France; EGM CNRS, Université Paris-Cité,Institut de Biologie Physico-Chimique, 13 rue Pierre et Marie Curie, 75005 Paris, France; Proteomics platform, FR550 Institut de Biologie Physico-Chimique, 13 rue Pierre et Marie Curie, 75005 Paris, France; Instituto de Tecnologia Química e Biológica António Xavier, Universidade Nova de Lisboa, 2780-157Oeiras, Portugal; Université Paris-Saclay, INRAE, AgroParisTech, Micalis Institute, 78350 Jouy-en-Josas, France; EGM CNRS, Université Paris-Cité,Institut de Biologie Physico-Chimique, 13 rue Pierre et Marie Curie, 75005 Paris, France

## Abstract

Faced with nutritional stress, some bacteria form endospores capable of enduring extreme conditions for long periods of time; yet the function of many proteins expressed during sporulation remains a mystery. We identify one such protein, KapD, as a 3′-exoribonuclease expressed under control of the mother cell-specific transcription factors SigE and SigK in *Bacillus subtilis*. KapD dynamically assembles over the spore surface through a direct interaction with the major crust protein CotY. KapD catalytic activity is essential for normal adhesiveness of spore surface layers. We identify the *sigK* mRNA as a key KapD substrate and and show that the stability of this transcript is regulated by CotY-mediated sequestration of KapD. SigK is tightly controlled through excision of a prophage-like element, transcriptional regulation and the removal of an inhibitory pro-sequence. Our findings uncover a fourth, post-transcriptional layer of control of *sigK* expression that couples late-stage gene expression in the mother cell to spore morphogenesis.

## Graphical Abstract

**Graphical Abstract:**
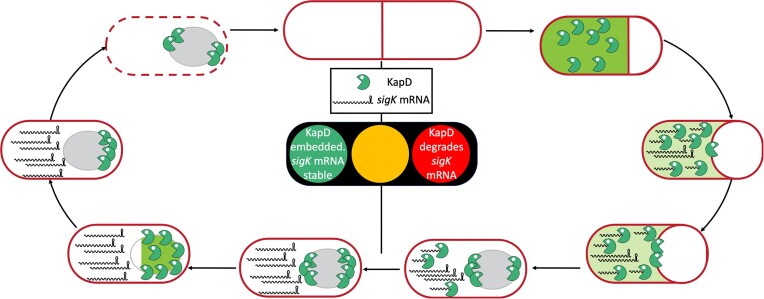

## Introduction

Two major families of Gram-positive bacteria, the Bacillaceae and Clostridiaceae, use an evolutionarily conserved mechanism to form metabolically dormant endospores. Such resilient life forms have been successfully revived from the gut of a >25-million-year-old bee fossilized in amber (1) and from a 250-million-year-old salt crystal (2). These bacteria include pathogens such as the agents of anthrax, botulism, tetanus and chronic intestinal diseases. The best studied of the spore-formers is *Bacillus subtilis*, which initiates this sophisticated developmental program upon encountering severe nutritional stress (for recent reviews, see (3,4)). During sporulation, the bacterial chromosome is first replicated, an asymmetric septum forms close to one pole of the cell and the newly replicated chromosome is partitioned into the smaller daughter cell compartment, called the forespore. The larger cell, known as the mother cell (MC), then engulfs the forespore and builds a multi-layered protective envelope around it. Finally, the MC undergoes lysis to release the mature spore. These different stages are identifiable microscopically, and the whole developmental process involves coordinated expression of several hundred genes and typically takes about 8 h to complete under laboratory conditions. It is coordinated by a cascade of spatially and temporally regulated RNA polymerase sigma factors (SigF followed by SigG in the forespore and SigE followed by SigK in the MC) that relay each other through cross-talk between the MC and the developing spore (5,6).

The protective structure built around the forespore consists of a peptidoglycan cortex and a proteinaceous coat composed of four surface layers: the basement/undercoat layer, the inner coat, the outer coat and an external glycoprotein crust. Although the inner and outer coat layers can be assembled independently of each other, assembly of the inner coat and crust layers are dependent on the subjacent basement and outer coat layers, respectively (6). The four surface layers are made up of at least 80 different proteins (5–8) that are synthesized by the MC and encase the surface of the developing spore in concentric shells (9). Correct surface layer formation is necessary for spore resistance to harsh environmental conditions such as heat, desiccation, radiation or chemicals, as well as for the survival in *B. subtilis* in the gut of its natural predators, such as *Caenorhabditis elegans* (10) or *Tetrahymena thermophila* (11). The general assembly pathway of the major spore coat proteins is well-understood, with a small number of morphogenetic proteins playing key roles in the initial targeting of the coat proteins to the surface layers and their subsequent encasement of the developing spore (8). The assembly of each layer is thought to be driven by the localization and polymerization of the specific morphogenetic proteins (e.g. SpoVM and SpoIVA, SafA, CotE and CotZ for the basement, inner coat, outer coat and crust layers, respectively), creating binding sites for the individual proteins that make up the different layers (6). Encasement proteins (e.g. SpoVID) are required for the formation of a full shell for the different layers. Without them, other coat proteins can initially localize to the correct layer, but fail to cover the surface completely, resulting in defective spores.

The mRNAs encoding the different protein components of the spore coat are typically expressed under SigE control early in sporulation and subsequently by SigK late in the developmental process (12). However, little if anything is known about the contribution of mRNA degradation by ribonucleases (RNases) to their overall expression levels. About two dozen RNases have been already identified in *B. subtilis* and their substrates are well characterized. However, there are still some orphan ribonucleases encoded by the genome of *B. subtilis* with no known function.

**Figure 1.**
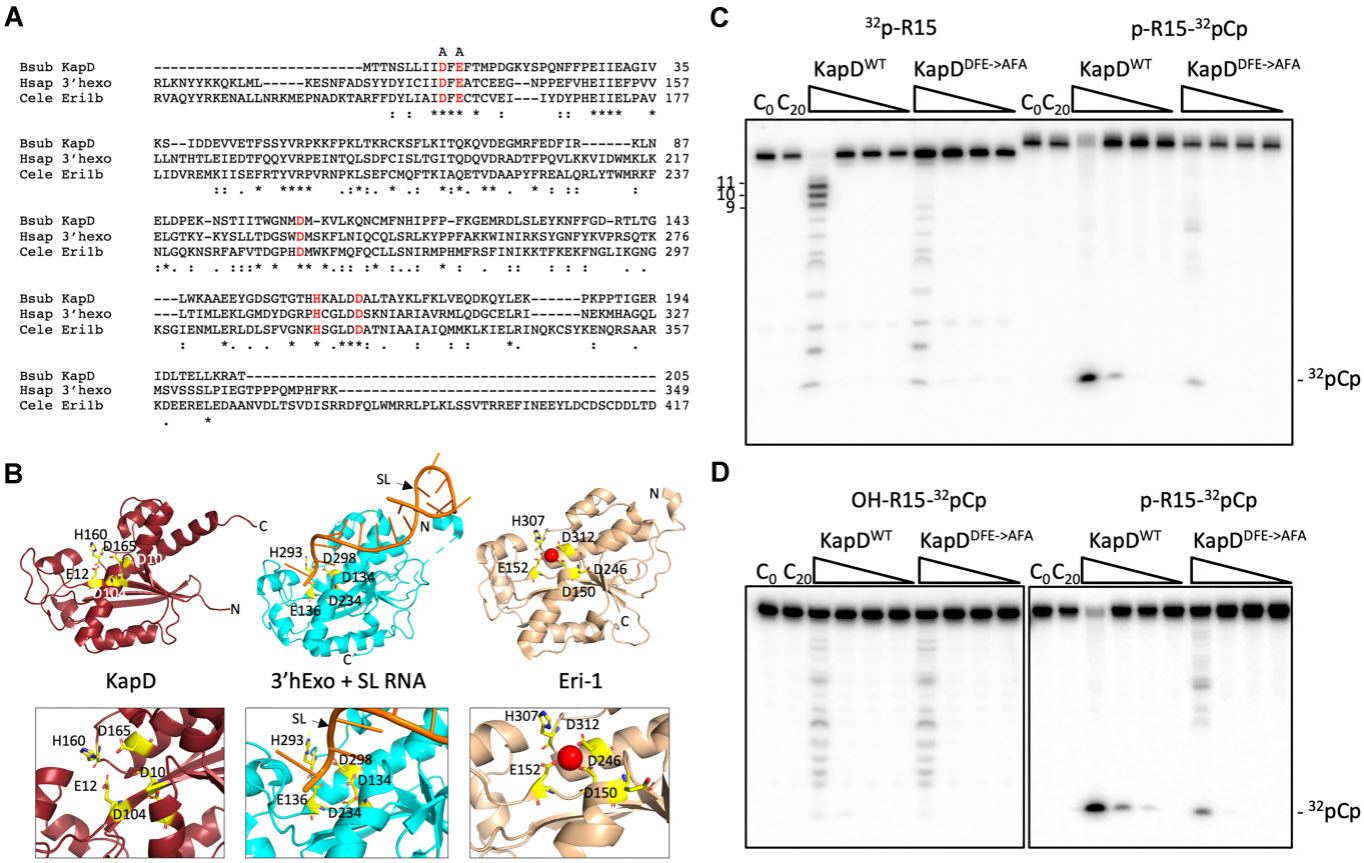
KapD is a sequence and structural homolog of 3′hExo and Eri-1 and has 5′-end-dependent 3′-exoribonuclease activity *in vitro*. (**A**) Sequence alignment (Clustal Omega) of *B. subtilis* KapD, human 3′-hExo (Hsap) and *C. elegans* Eri-1b (Cele Eri-1b). (*) shows identical amino acids, (:) designates similar amino acids and (.) indicates similar groups of amino acids. Amino acids that form the DEDDh motif are shown in red. The DE residues changed to A to create a catalytic mutant are indicated. (**B**) Comparison of Alphafold2 model(82) of *B. subtilis* KapD with human 3′hExo (4qoz) bound to histone mRNA stem-loop (SL, orange) and human Eri-1 exoribonuclease 3 (2xri); the red sphere represents a Mg^2+^ ion. Note that the KapD model omits a C-terminal extension of 18 residues. In the model of human 3′hExo, the stem-loop binding protein is not included. In all cases, the residues highlighted in yellow form the active site and correspond to DEDDh motif shown in panel A. The numbers refer to the sequence included in the models. The N- and C-termini are indicated. The bottom panels show enlarged views of the active site region of each protein. (**C**) *In vitro* activity assay performed on the synthetic R15 RNA (CCR018, Supplementary Table S3), labeled at either the 5′ (left) or 3′ end (right). Reactions were performed with a 10-fold serial dilution of KapD from 10^−1^ μg/μl to 10^−4^ μg/μl final concentration, represented by right angled triangles. The liberation of ^32^pCp is evidence of a 3′-5′ exonuclease activity, while the accumulation of 9–11 nt intermediates, suggests the enzymes loses processivity once the RNA is reduced to this size. Despite a two-step purification of KapD (see ‘Materials and methods’), a background ladder of RNase activity is observed from a contaminant in preparations of both the WT and KapD catalytic mutant. (**D**) *In-vitro* activity assay performed on the synthetic RNA R15, labeled at the 3′ end with ^32^pCp and bearing either a 5′-phosphate (p) or 5′-hydroxyl (OH).

One such orphan ribonuclease is encoded by the *kapD* gene and is expressed principally during sporulation (12–15), but its role in this process remains a mystery. KapD is a member of the DEDDh family of 3′-exoribonucleases, closely related to the Eri-1 ribonuclease, involved in the degradation of siRNAs and 5.8S rRNA processing in *C. elegans* (16,17) and to human 3′-hExo, characterized for its implication in the maturation and degradation of histone encoding mRNAs (18) (Figure 1A, B). KapD stands for kinase associated protein D and is annotated as an inhibitor of the KinA pathway that signals the initiation of sporulation, but the experimental basis for this annotation has not been published. Here, we show that KapD is an active 3′-exoribonuclease *in vitro* and that its expression is controlled by the MC-specific sigma factors, SigE and SigK. Fluorescence microscopy experiments show that KapD dynamically assembles onto the surface of the developing spore, and transmission electron microscopy (TEM) experiments show that the catalytic activity of KapD, rather than simply its presence, is critical for correct spore morphology. Finally, we show that the *sigK* mRNA is itself a substrate for KapD and its half-life is regulated by KapD sequestration in the crust by direct protein–protein interaction with the major crust morphogenetic protein, CotY. The timing and levels of SigK expression and activity are subject to multilevel regulation (19) involving a genome rearrangement via excision of the *skin* prophage-like element that interrupts its coding sequence (20,21), direct transcriptional regulation by SigE, GerR, SpoIIID and GerE (12) and post-translational activation by proteolytic removal of a 19-amino acid inhibitory pro-sequence from its N-terminus (22–24). Post-transcriptional regulation by KapD and CotY adds yet a fourth regulatory layer to the control of this key sigma factor, which is essential for correct spore morphogenesis.

## Materials and methods

### *B. subtilis* strains

All strains were derived from the laboratory strain *B. subtilis* W168 (SSB1002), except when otherwise specified. A list of all strains used is provided in Supplementary Table S2. Details of new strains and plasmid constructs are provided below. Oligonucleotides used for these constructs are given in Supplementary Table S3.

### Construction of *ΔkapD* strains

Strain CCB567 (*kapD::ery*) was constructed by three-fragment overlapping polymerase chain reaction (PCR) with oligo pairs CC1354/1355 (*kapD* front), CC1365/66 (*ery*) and CC1356/1357 (*kapD* back); then reamplified with nested oligos CC1370/1371. The template for amplification of the *ery* cassette was plasmid pDG641 (25). The resulting PCR fragment was transformed into strain SSB1002 and verified by PCR amplification with oligos CC1354 and CC1357.

Strain CCB607 (*kapD::spc ery-ter*) was constructed by overlapping PCR with oligo pairs CC1354/1436 (*kapD* front), CC1437/1438 (*spc*), CC1439/1366 (*ery-ter*) and CC1356/1357 (*kapD* back), in two steps. The *spc* and *ery* terminator (*ery-ter*) fragments were first combined with oligos (CC1437/1366), then all three fragments were combined and reamplified with oligos CC1354/1357. The templates for amplification of the *spc* cassette and the *ery* terminator were plasmids pDG1727 and pDG641 (25), respectively. The resulting PCR fragment was transformed into strain SSB1002 with selection for spectinomycin resistance and verified by PCR amplification with oligos CC1468/CC1010 and CC1457/1458 and sequencing of surrounding chromosomal context. A silent mutation CAT→CAC (His75) is present in the *kapB* gene, which was considered acceptable for the purposes of these studies.

### Construction of the *kapD^AFA^* catalytic mutant

A markerless *kapD^AFA^* catalytic mutant was constructed by cloning an overlapping PCR fragment (oligo pairs CC1375/2071 and CC1376/2070, reamplified with CC1375/1376) into pMAD (26) digested with SalI/EcoRI. The resulting plasmid pMAD-KapD (DFE→AFA) (plasmid number 834) containing the D10A and E12A mutations was confirmed by sequencing. Pop in/pop out of this construct was performed according to (26). Correct replacement resulted in the destruction of a BstBI restriction site allowing a first screen by PCR amplification of the *kapD* locus (oligos CC1375/1376) and digestion by BstBI, followed by sequencing for confirmation. The resulting strain was called CCB1251.

### Construction of *kapD* complementation/overexpression strains

Two *kapD* complementation strains were constructed that differ by the presence of *Escherichia coli rrnB t1t2* transcription terminators to insulate from potential antisense transcription from the promoter of the *thrC* gene into which the of a copy of the *kapD* gene was inserted in the inverse orientation. In these constructs, a region extending from 170 nts upstream to 27 nts downstream of the *kapD sigK* promoter was fused 170 nts upstream of the native *sigE* promoter and associated *kapD* gene by overlapping PCR. The *sigK* promoter was amplified using oligos CC2605/2588 and the *sigE* promoter + *kapD* gene was amplified using oligos CC2589/2590. Oligo CC2590 contained an integrated terminator hairpin sequence to provide a defined and stable 3′ end to the *kapD* mRNA. The two PCR products were reamplified with oligos CC2605 and CC2950, digested with EcoRI and cloned in plasmid pDG1664 for integration into the *thrC* locus (27). The resulting plasmid, pDG1664-*kapD* (plasmid number 885), was linearized with XhoI and transformed into *B. subtilis* wild-type (WT, SSB1002), *kapD^AFA^* (CCB1251) and *ΔkapD* (CCB607) mutant strains, with selection for macrolide-lincosamide-streptogramin (MLS) resistance and screening for threonine auxotrophy. The resulting strains were called CCB1373, CC1374 and CCB1375, respectively. In the insulated derivative, the *E. coli rrnBt1t2* terminators were amplified using oligo pair CC3243/CC3244 and plasmid pMUTIN-4M as template (28), the *kapD* gene with associated promoters and terminator locus was amplified using oligos CC3245/CC3246 and pDG1664-*kapD* as template, and the overlapping product reamplified using oligo pair CC3243/CC3246. Plasmid pDG1664 was digested with EcoRI and the insert cloned with the NEBuilder Hifi DNA Assembly Mix (NEB). The resulting plasmid, pDG1664-*rrnBt1t2-kapD* (plasmid number 992), was linearized with NcoI and transformed into the *ΔkapD* strain CCB607 with the selection for MLS resistance. The resulting strain was called CCB1744.

### Construction of GFP fusions

The KapD-GFP translational fusion (pCVO119m-KapD-GFP; plasmid number 671) was made by PCR amplification of a portion of the genome downstream of the *kapD* ORF using oligo pair CC1675/CC1676 and the 3′ half of the *kapD* ORF using oligo pair CC1674/CC074, cleaving the resulting PCR fragments with SpeI/BamHI and BamHI/SalI, respectively, and ligating both fragments together with vector pCVO119m (a derivative of pCVO119 (29), containing the A206K mutation to create monomeric GFP (Carballido-Lopez lab collection)), cleaved with SpeI and SalI. The resulting plasmid was sequenced, linearized with BamHI and inserted into the *kapD* locus of *B. subtilis* strain SSB1002 by double recombination to yield strain CCB786.

### Construction of KapD-6xHis and CotY-3xFlag overexpression plasmids

Plasmid pET28-KapD-His (plasmid number 313) was constructed by PCR, amplifying the *kapD* gene with oligos CC073/074 and cloning in plasmid pET28a (Novagen) digested with EcoRI and SalI. Plasmid pET28-KapD^AFA^-His (plasmid number 775) was made by site-directed mutagenesis of pET28-KapD-His using oligos (CC2070/2071). The plasmids were verified by sequencing and transformed into BL21 CodonPlus RIL for overexpression, yielding strains CCE095 and CCE273, respectively.

pET28-CotY-Flag (plasmid number 770) was made by amplifying the *cotY* gene by PCR using oligos (CC2058/2059), combining with two complementary oligos encoding the 3xFlag (CC2054/2055) and cloning in pET28a digested with EcoRI and HindIII. The plasmid was verified by sequencing and transformed into BL21 CodonPlus RIL for over expression, yielding strain CCE275.

### KapD protein purification

WT and catalytic mutant (KapD^AFA^) were overexpressed in *E. coli* BL21 CodonPlus RIL as C-terminal His-tagged proteins. Strains were grown in 400 mL LB media with antibiotics for plasmid selection, until the OD_600_ reached 0.6. Proteins were induced with a final concentration of 0.5 mM IPTG, for 3 h. The pellets were washed with 25 mL cold TE-NaCl [10 mM Tris-HCl pH 8, 1 mM EDTA, 0.1 M NaCl], centrifuged 8000 rpm, 10 min and 4°C (Sorval JA-12 rotor). The pellet was resuspended in 10 mL equilibrium buffer [20 mM Tris-HCl pH 8.8, 0.5 M NaCl, 10% glycerol, 0.1% Triton-X100], protease inhibitor cocktail (Roche) and 1 μl/mL DNase I [10 mg/mL] (Sigma). The pellets were lysed by 2–3 passages in a French Press (20 000 p.s.i). Lysates were centrifuged at 10 000 rpm for 20 min at 4°C (Sorval JA-20 rotor), and a final concentration of 1 mM imidazole was added before adsorbing to the His-Trap column (GE Healthcare), pre-equilibrated with equilibrium buffer. The column was washed with 10 column volumes of equilibrium buffer and 10 column volumes of washing buffer I_20_ [20 mM Tris-HCl pH 8.8, 20 mM imidazole, 0.3 M NaCl], then in same buffer with 40 mM imidazole (I_40_) and, finally, eluted with elution buffer [20 mM Tris-HCl pH 8.8, 500 mM imidazole, 0.3 M NaCl], in 1 mL fractions. Peak fractions were identified by Bradford Assay (Biorad), pooled and dialysed against buffer [20 mM Tris-HCl pH 8.8, 150 mM NaCl, 10% glycerol, 0.5 mM DTT]. The dialysed material (∼2 mL) was applied to a preparative gel filtration column HighLoad Superdex 75 16/60 (GE Healthcare) equilibrated in the same buffer. Four-mL fractions were collected and peak fractions pooled. Pooled fractions were applied to a MonoQ HR 5/5 anion exchange column (GE Healthcare), pre-equilibrated in buffer [20 mM Tris-HCl pH 8.8, 50 mM NaCl, 10% glycerol]. The column was washed with 10 column volumes of the same buffer. KapD proteins were eluted in 1 mL fractions using a 50 mM to 2 M NaCl gradient in the same buffer. Proteins were concentrated using spin columns (Amicon ultra 10 KDa) and stored at −80°C before use. The purity of each purification step was verified by Coomassie-stained sodiumdodecyl sulphate-polyacrylamide gel electrophoresis (SDS-PAGE).

### 5′ and 3′ labeling of RNA substrates

The synthetic RNA substrate used for *in-vitro* KapD activity assays was a 15-nt RNA (R15), starting with uridine, adenine or guanidine (CCR018, CCR034 or CCR036, respectively; see Supplementary Table S3). For 5′ labeling, 40 μM R15 oligo was labeled using 3 μl of adenosine triphosphate (ATP) [γ-^32^P], 2 μL 10x T4 PNK Buffer (NEB) and 2 μL of T4 PNK kinase (NEB) in a final volume of 20 μL and incubated for 40 min at 37°C. For 3′ labeling, 100 μM RNA was labeled using 2.5 μL of DMSO 100%, 1.25 μL of 10 mM ATP, 2.5 μL of 10x RNA ligase buffer (NEB), 1.25 μL of rRNasin 40 u/μl (Promega), 2 μL of RNA ligase (NEB) and 3 μL of pCp [5′-^32^P] in a final volume of 25 μl incubated at overnight 4°C. The 3′ end labeled R15 was phosphorylated at its 5′ end with cold ATP by adding 1 μL of 10x T4 PNK Buffer (NEB), 3.5 μL of 10 mM cold ATP and 3 μL of T4 PNK enzyme (NEB) in a final volume of 35 μL for 40 min at 37°C. Labeled RNAs were gel-purified by running for 1 h at 60W on a 20% polyacrylamide-7M urea gel, the corresponding bands were excised and eluted for at least 4 h in 400 μL of 0.3M sodium acetate. The RNA was ethanol precipitated and resuspended in 20–30 μL nuclease-free water. To generate a 5′ tri-phosphorylated RNA, a double-stranded DNA template containing a T7 promoter sequence and a 16-nt coding sequence was generated by annealing two complementary oligonucleotides (CC2524 and CC2525, see Supplementary Table S3). The oligonucleotides (10 uM each) were denatured at 95°C for 5 min in a buffer containing 10 mM Tris pH8, 50 mM NaCl and 1 mM EDTA and slowly cooled to room temperature for an hour. The template was transcribed using the MEGAshortscript™ T7 Transcription Kit (ThermoFisher Scientific) and 3′-end labeled using p^32^Cp, as described above. To generate the mono-phosphorylated derivative, the transcribed RNA was dephosphorylated at 37°C for 1 h using QuickCIP (NEB) in CutSmart buffer (NEB). The RNA was then 5′-phosphorylated as described above.

### KapD *in-vitro* activity assays

*In-vitro* activity tests were done in 10 μl volume, containing 0.5 μL radiolabeled RNA (3′ or 5′ end labeled), 2 μL 5x KapD reaction buffer [50 mM Tris-HCl pH 8.8, 8 mM MgCl_2_, 100 mM NaCl, 0.5 mM DTT], 6.5 μL nuclease-free water and 1 μL KapD protein (10-fold dilutions from 1 mg/mL to 10^−3^ mg/mL). For control reactions, 1 μL nuclease-free water was used instead of the protein. The reactions were incubated 20 min at 37°C and stopped by adding 10 μL 2xRNA loading dye (Ambion). Eight μL of the reaction was denatured 5 min at 90°C and run on a 20% polyacrylamide gel for 1 h at 60W in 1x TBE buffer. The gel was vacuum dried for 1 h at 80°C and exposed to a phosphor-Imager screen.

### Sporulation cultures

Sporulation was either induced by exhaustion in Difco sporulation medium (DSM) supplemented with 1 M Ca(NO_3_)_2,_ 0.01 mM MnCl_2_ and 1 mM FeSO_4_ (30) or by the the resuspension method of Sterlini and Mandelstam (31). Cultures were typically sampled every hour for 8 h (T0 to T8) after the inflexion point in DSM (defined as the initiation of sporulation or T0) or after resuspension in sporulation medium. For resuspension experiments involving the complementation construct in *thrC*, we added threonine (50 μg/mL) to all cultures. For time-lapse Lattice SIM imaging, sporulation was performed in microcolonies, as described previously (32). Briefly, cells were collected from sporulation cultures, labeled with SNAP-Cell^®^ TMR-Star (New England Biolabs) for 30 min at 37°C in the dark at a final concentration of 250 nM and directly applied to a gene frame containing a 1.6% agarose pad in resuspension medium. Then, the microscopic slide was incubated overnight in the dark to allow microcolony development and their entry in sporulation. Time-lapse acquisitions started upon the detection of KapD-GFP signal.

### Epifluorescence microscopy

Sporulation was initiated either by exhaustion in DSM (Figure 3A; first four panels) or the resuspension method (Figure 3A (phase grey), 3C and S2) (31). For the exhaustion method, overnight cultures from single colonies of *B. subtilis* strains were diluted in DSM at OD_600_ 0.01 and grown at 37°C. Samples were taken from 2 to 4 h after the inflection point of the growth (OD_600_ ∼1.8). For the resuspension method, overnight cultures from single colonies of *B. subtilis* strains, grown in a defined rich medium (CH) were diluted 1:50 in CH up to OD 1 and re-suspended in a defined poor medium (SM). The time of resuspension corresponds to time T0 of sporulation. Samples were taken every hour from T1 to T6. Cells were mounted on 1.2% agarose pads and stained with FM4–64 (Molecular Probes), to visualize the cell membrane, and DAPI (Thermofisher) to visualize the nucleoid. Fluorescence microscopy was performed on a Nikon Eclipse Ti (100 FluoPlan objective with an aperture of 1.30 and an ORCA R2 camera (Hamamatsu)) equipped with an environmental chamber maintained at 37°C. Image processing was performed using Fiji software.

### Dual-color lattice SIM^2^

Lattice SIM imaging, TMR-star labeling and mounting of samples were conducted as previously described (32) using an Elyra 7 AxioObserver (Zeiss) inverted microscope yielding a final pixel size of 64.5 nm for raw images. Fluorescence was excited using 488 nm (100 mW) and 561 nm (100 mW) laser beams at 40% and 20% of maximal output power, respectively. For snapshots, 15 phases were acquired. Exposure time per phase was 80 and 20 ms for 488 and 561 nm laser, respectively. For single-cell time-lapse acquisitions, 13 phases were acquired with an exposure time reduced to 60 ms for the 488-nm laser beam. Temperature was maintained at 30°C during acquisition. Lattice SIM image reconstruction was performed using the Zen Software (Zeiss, black edition) and the nonlinear dual iterative reconstruction algorithm SIM² with general settings as previously described (33), yielding a final pixel size of 16 nm for reconstructed images. Quantifications of KapD-GFP fluorescence intensity in the MC was performed on pseudo-widefield images prior to SIM image reconstruction. A polygonal region of interest (ROI) was adjusted using the Fiji polygon tool in the center of the MC cytoplasm of the sporulating cell. The mean intensity of the ROI was recorded and the value of the background signal was subtracted from each measurement, for the indicated numbers of sporulating cells.

Statistical analyses were performed using Rstudio version 4.1.1 for PC. Data are represented with box-plots showing the interquartile range (IQR; 25th and 75th percentile). The upper whisker extends from the upper hinge to the largest value no further than 1.5 × IQR from the hinge and the lower whisker extends from the hinge to the smallest value at most 1.5 × IQR of the hinge.

### Fluorescence recovery after photo-bleaching (FRAP)

Sporulating cells collected 4 and 5.5 h after resuspension in sporulation medium were mounted on glass slides coated with resuspension medium in 1.6% agarose and imaged using Nikon Ti-E fitted with a 100x/1.49 NA oil objective Apo TIRF, an EMCCD Andor iXon 897 camera (Oxford Instruments) and stabilized with Perfect Focus, at 30°C. Samples were first imaged by brightfield. Then, images were taken every 2 s for 1 min using a 488-nm laser at 30% power with 100-ms exposure. FRAP was performed using MetaMorph the iLAS² module. Fluorescence was bleached with a pulse from a 488-nm laser set to 60% power on an area 500 pixels in diameter. Time-lapse image sequences were stabilized using the Fiji plugin ‘StackReg’, using transformation as « rigid body ». Quantification was performed as described elsewhere (34). Briefly, we calculated the normalized fluorescence recovery by measuring the relative intensity of the bleached area (MCP cap signal in engulfed forespores or MCD pole of refractile forespores) to the signal in an unbleached cell detected in the same field of view. The pre-bleaching ratio was defined as 1 and the ratio immediately after the bleaching event as 0.

### Yeast-two-hybrid screening and specificity assay

A pGAD-expressed *B. subtilis* genomic library in yeast was screened with the pGBDU bait vector expressing KapD in yeast, as previously described (35,36). After mating on rich medium, diploid individuals were selected for interacting phenotypes on defined media lacking leucine, uracil and histidine (-LUH) or adenine (-LUA). Prey candidates were identified by PCR amplification and sequencing of the pGAD plasmid inserts. To remove false-positive interactions, a specificity assay was done. Prey fragments were recloned in pGAD in yeast by gap-repair (37) and resequenced, and a new interaction assay was performed using a positive control and the empty vector to test for autoactivation. Interactions were considered specific when they were reproduced with their original bait protein. Specific prey sequences were rechecked.

### KapD and CotY co-purification

*E. coli* strains overexpressing KapD-6xHis (CCE095) and CotY-3xFLAG (CCE275) were grown and induced with IPTG, as described above, except that expression of CotY-3xFLAG was induced for 4–5 h. The two pellets were mixed and lysed together by French Press in the presence or absence of RNase A (10 μg/mL). After an initial purification step on a His-Trap column (GE Healthcare) as described above, the peak protein fractions were pooled and injected onto an analytical gel filtration column Superdex 200 10/300 GL column (GE Healthcare) equilibrated in [20 mM Tris-HCl pH 8.8, 150 mM NaCl, 10% glycerol]. Around 1 mL fractions were collected and 20 uL deposited on an SDS-PAGE gel (12%) for Western blot analysis.

### Western blot analysis

For Western blots, proteins were transferred to Hybond-C membranes (GE Healthware) by electro-transfer. Membranes were blocked in PBS-T [1X PBS, 0.1% Tween 20] and 5% low-fat powder milk solution. Primary antibodies were used at the following dilutions: KapD 1/1000 (Spore Coat Western blots), KapD-His 1/20 000 (Co-purification) and CotY-FLAG (1/1000). A secondary peroxidase-conjugated anti-rabbit antibody was used at a concentration of 1/10 000. Western blots were developed with using chemi-luminescence reagents (GE Healthcare).

### TEM analysis

Spores were purified from DSM cultures (exhaustion method) 18 hours after initiation of sporulation and processed for thin sectioning TEM essentially as described (38). Samples were imaged on a Hitachi H-7650 microscope equipped with an AMT digital camera, operated at 100 keV.

### Heat resistance and germination assays

Heat resistance and germination assays were performed according to Nicholson and Cutting (39) with minor modifications as described in the legend to Supplementary Table S4.

### Mass-spectrometry analysis of *B. subtilis* spore coat composition

Strains were grown in DMS (exhaustion method) supplemented with 1 M Ca(NO_3_)_2,_ 0.01 mM MnCl_2_ and 1 mM FeSO_4_ for 36 h. Spores were purified by a two-step gradient of Gastrografin as described in (40). Briefly, after a 36-h culture, cells were harvested and washed three times with cold RNase/DNase free water. The pellet was resuspended in the same volume of ice-cold water and incubated at 4°C for 24 h for the cells to lyse. Next, the pellet was washed three times with water and resuspended in 1 mL 20% Gastrografin solution (Bayer Schering Pharma). The resuspended spores were layered on top of a 50% Gastrografin solution (20 mL) and centrifuged at 10 000 g at 4°C for 30 min in a bench-top centrifuge (Eppendorf). The purified spores in the pellet were recovered and washed five times with ice-cold water to eliminate traces of the Gastrografin solution.

Total coat proteins were extracted by boiling the spores (equivalent to an optical density at 580 nm of 450) for 8 min in an extraction buffer [125 mM Tris pH 6.8, 4% SDS, 10% (v/v) b-mercaptoethanol, 1 mM DTT, 0.05% bromophenol blue, 10% glycerol] as described in (Henriques and Moran, 2000). The mixture was centrifuged 5 min at maximum speed and 5 μL of supernatant, corresponding to about 30 μg (450 OD units) extracted protein were loaded onto a 15% SDS-PAGE gel and run to just past the stacking part of the gel. The proteins were cut from the gel, digested by trypsin and applied to a tandem NanoEasy1000, Q-exactive (Thermoscientific) mass spectrometer. Spores from WT and Δ*kapD* strains were isolated as five biological replicates, each injected twice for analysis. Statistical analysis was done by using a t-student test, with *P* < 0.05 chosen as the cut-off. For Western blot analysis of spore coat composition, spores were boiled for 20 min in extraction buffer, and 10 μL were loaded on 15% SDS-PAGE gels.

### Northern blots

For Northern blot analysis, RNA was typically isolated from 2 mL of sporulating cultures by RNASnap^TM^ (41) or the phenol/glass beads method (42) and the equivalent of 5 μg total RNA was loaded onto a 1% agarose gel and run in 1x TBE solution as described in (43). For half-life measurements rifampicin was added at 150 μg/mL before RNA isolation. The list of all the probes used are listed in the Supplementary Table S3.

## Results

### KapD is a 5′-sensitive 3′-5′ exoribonuclease *in vitro*

Given its homology to the known DEDDh-family of 3′-exoribonucleases, 3′hExo and Eri-1, we first sought to confirm that KapD indeed had 3′-exonuclease activity *in vitro*. For this purpose, we used a 15-nucleotide (nt) synthetic oligoribonucleotide we call R15 (UGGUGGUGGAUCCCG) that we previously used to characterize the directionality of the exoribonuclease RNase J1 (44) and an optimized buffer for KapD activity (See ‘Materials and methods’). R15 was ^32^P-labeled at either its 5′ or 3′ end, using γ-^32^P-ATP and T4 polynucleotide kinase (to generate ^32^p-R15), or α-^32^P-pCp and RNA ligase (to generate p-R15-^32^pCp), respectively, and incubated with a 10-fold serial dilution of purified KapD. To directly compare the two reactions, the 3′-labeled oligoribonucleotide was also 5′-phosphorylated. Digestion of 3′-labeled R15 with KapD yielded only ^32^pCp, as expected for a 3′-exoribonuclease (Figure 1C), while digestion of the 5′-labeled R15 yielded primarily 9-11-nt end-products. This suggests that KapD behaves as a processive 3′-exoribonuclease until RNAs reach a critical size of 9–11 nts and becomes distributive thereafter. A catalytic mutant, with the conserved DxE motif ^10^DFE^12^ (Figure 1A) changed to ^10^AFA^12^ (KapD^AFA^), showed >10-fold reduction in the release of pCp (Figure 1C), confirming that the observed activity is associated with KapD.

5′ monophosphate groups have previously been shown to stimulate the endoribonuclease activity of some bacterial RNases, e.g. RNase E (45) and RNase Y (46) and to be the preferred 5′-moiety on the RNA substrates of 5′-exoribonucleases such RNase J1 (44) or the eukaryotic Xrn1 (47). Although the identity of the 5′-moiety was not previously known to impact the activity of 3′-exoribonucleases, we nonetheless asked whether the 5′-phosphate group on the p-R15-^32^pCp substrate had an impact on KapD activity. To our surprise, KapD’s ability to attack the 3′ end of an RNA bearing a 5′-hydroxyl group instead of a 5′-monophosphate moiety was reduced by 1–2 orders of magnitude (Figure 1D). This suggests that KapD has a binding site for a 5′-monophosphate group that can stimulate the 3′-attack of its substrates. Stimulation by a 5′-monophosphate was observed with the same oligonucleotide where the 5′ nucleotide was changed to A or G (Supplementary Figure S1A), suggesting the phenomenon is independent of the identity of the first nucleotide. A 5′-mono-phosphate was also preferred over a 5′-triphosphate, which showed very little reactivity with KapD (Supplementary Figure S1B).

### The *kapD* gene is expressed under SigE and SigK control during sporulation

**Figure 2.**
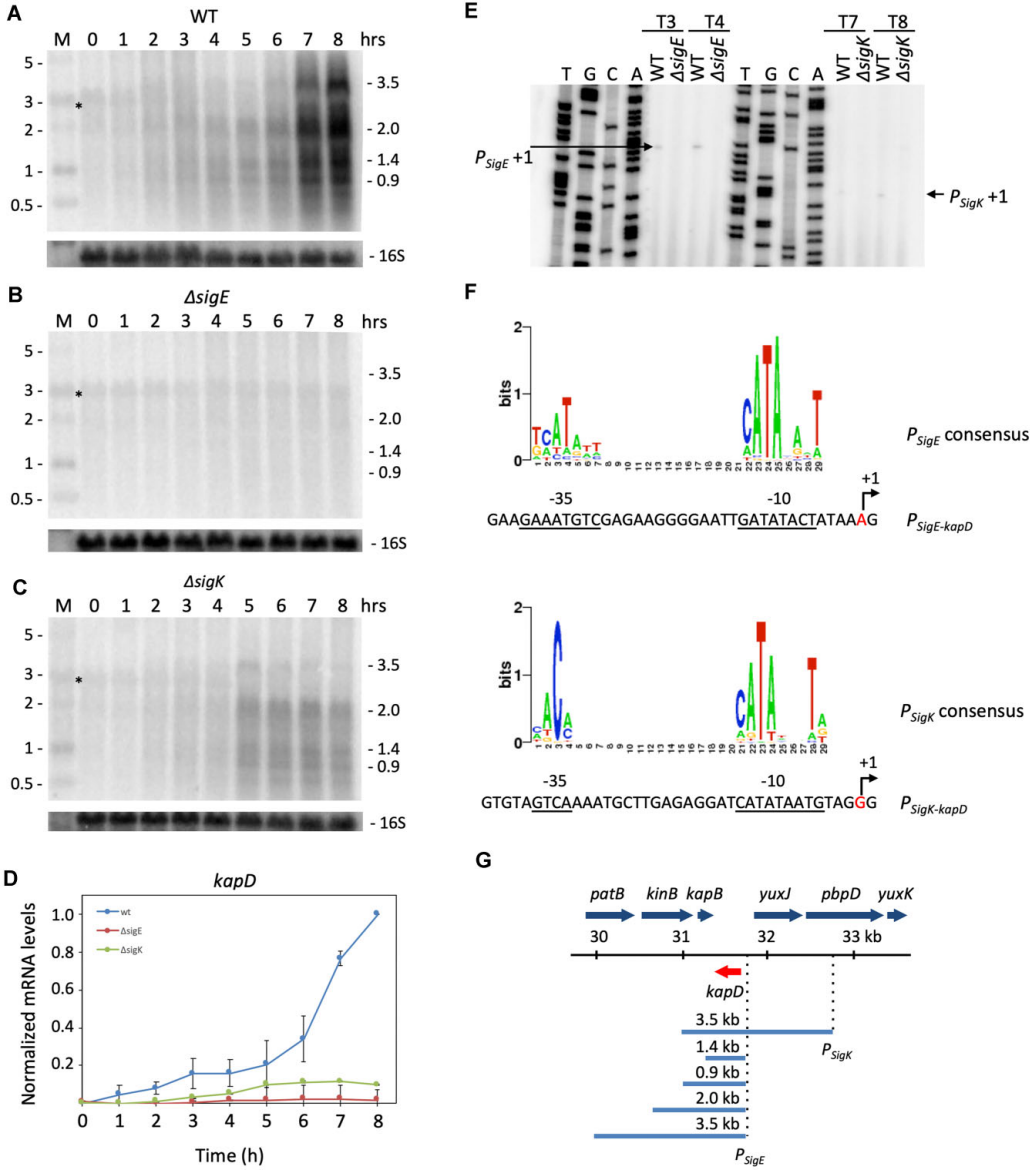
Expression of the *kapD* mRNA during sporulation is dependent on Sigma E and Sigma K. Northern blot probed with a *kapD* riboprobe in (**A**) WT, (**B**) *ΔsigE* and (**C**) *ΔsigK* strains at different times after the onset of sporulation (hour 0). An RNA size-standard is shown in the lane labeled M (in kb). Membranes were reprobed for 16S rRNA as a loading control (oligo CC058). The band labeled with an asterisk likely corresponds to non-specific hybridization of the *kapD* probe to 23S rRNA. (**D**) Quantification of two independent experiments exemplifed by those shown in panels A–C. Signals were normalized to the maximal value for the three membranes. Error bars represent standard deviation. (**E**) Primer extension assay showing detection of 5′ ends close to the SigE and SigK consensus sequences (**F**) at various times (T3, T4 and T7, T8) after the onset of sporulation in WT, *ΔsigE* or *ΔsigK* strains. (**G**) Genetic organization of the *kapD* region of the chromosome and summary of major *kapD* transcripts identified by Northern blot and primer extension assays. The *kapD* gene is highlighted in red.

Early studies showed that *kapD* was a member of the SigE and SigK regulons suggesting a potential role in sporulation (12,14). *kapD* expression was also shown to be repressed by the transcriptional regulator GerR, a fellow member of the SigE regulon, and to be activated by GerE, a fellow member of the SigK regulon. Tiling array experiments performed under >100 different growth conditions confirmed that the *kapD* gene is primarily expressed during sporulation in *B. subtilis*, with expression beginning around 3 h (T3) after resuspension into sporulation medium and continuing to rise until the end of the program at T8 (13). Expression was predicted to be driven by a SigE-dependent promoter just upstream of the *kapD* ORF and a SigK-dependent promoter about 2 kb further upstream, antisense to the *pbpD* gene. We confirmed this expression profile by Northern blot using a riboprobe complementary to the *kapD* mRNA. Four transcripts ∼3.5, ∼2.0, ∼1.4 and ∼0.9 kb in length began to appear around T2/T3, gradually increasing in intensity before a strong burst of expression occurred at T7 and T8 (Figure 2A,D). In a *ΔsigE* mutant, no *kapD* expression whatsoever was observed (Figure 2B,D), while in a *ΔsigK* mutant, the late burst in expression was absent (Figure 2C,D). This is consistent with the hypothesis that SigK drives the late burst in *kapD* expression while SigE (which is also required for SigK expression) promotes the expression early in sporulation.

We confirmed that *kapD* expression originated from the predicted promoter regions by primer extension assay. A 5′ end that mapped just downstream of the predicted SigE promoter was observed at T3 and T4 in a WT strain, but was absent in a *ΔsigE* mutant (Figure 2E,F). Similarly, a SigK-dependent 5′ end was detected at T7 and T8 just downstream of the sequence resembling the SigK consensus sequence (Figure 2E,F). The fact that the SigE- and SigK-dependent transcripts have similar sizes on Northern blots (Figure 2A), suggests that the transcripts originating at the SigK promoter may be processed near the SigE start site. No obvious terminator structures account for the 3′ ends of these mRNAs, suggesting that the 3′ ends may also be the result of processing events and/or Rho-dependent termination (13). A global map of *kapD* containing transcripts from this region, based on the Northern blot and primer extension data, is shown in Figure 2G.

### *KapD* is expressed in the MC and localizes similarly to spore coat proteins

SigE and SigK are expressed in the MC during the sporulation process. To confirm that KapD expression and localization occur in the MC, we made a translational KapD-GFP fusion at the native *kapD* locus, where we fused the monomeric GFP coding sequence in frame with the full *kapD* coding sequence (see below for data that shows that the KapD-GFP protein fusion is fully functional). We then performed fluorescence microscopy experiments on strains carrying this fusion, coupled with FM4-64 staining of membranes and phase contrast microscopy, which are traditionally used to reveal morphological markers of the early sporulation stages (48,49). FM4-64 allows assessment of the first stages of sporulation from asymmetric septation through to the end of engulfment. After engulfment completion, the forespore becomes fully intracellular and its membranes are no longer accessible to the membrane dye. At this point, phase-contrast microscopy then allows monitoring the appearance of refractile phase dark, phase gray and phase bright spores, corresponding to early, intermediate and late stages of cortex maturation, principally.

**Figure 3.**
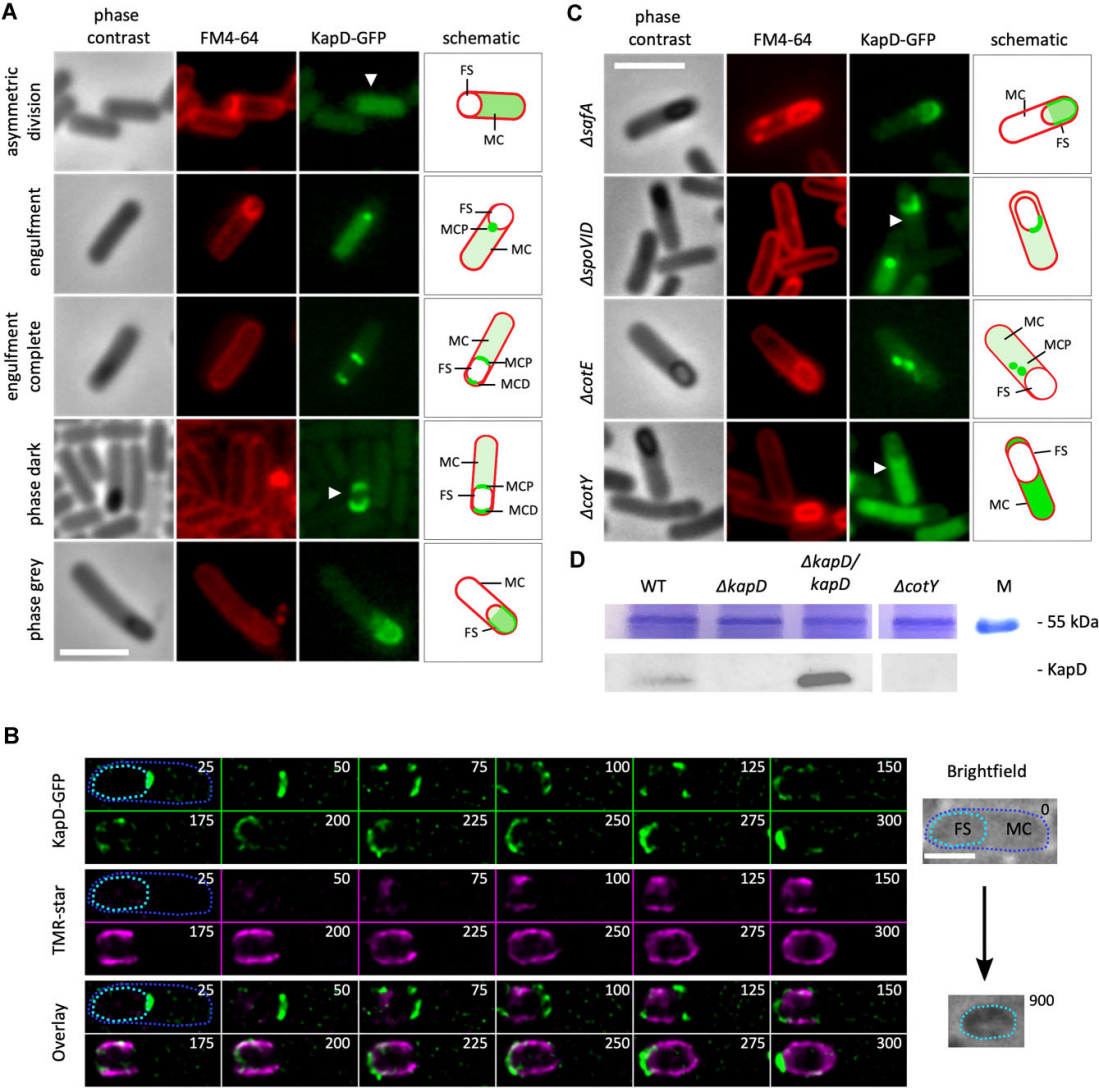
KapD is first expressed in the MC before localizing to the spore coat in a CotY-dependent manner. (**A**) Phase contrast and fluorescence microscopy images of cells containing the translational KapD-GFP fusion. Membranes were stained with FM4-64 (red). Representative cells are shown at different stages of sporulation (asymmetric division, during engulfment, engulfment completion, phase dark and phase gray). Scale bar, 3 μm. A schematic representation of the cell is shown in the right panel. FS, forespore; MC, mother cell; MCP and MCD, MCP and distal poles, respectively. (**B**) Single cell time-lapse of KapD-GFP (green) and TMR-star (pink) SIM^2^ super-resolved localization. Images are shown at 25-min intervals with times indicated on each panel. Arrowheads show widening of MCP cap, disappearance of KapD from the center of the MCP cap, appearance of horseshoes and retraction to small MCD cap, as described in text. Brightfield images show the cell before (time 0) and after (time 900 min) time-lapse imaging, showing the outline of MC (dark blue dotted line) and the position of future spore (light blue dotted line), and confirming the production of a refractile spore at the end of the experiment. FS, forespore; MC, mother cell. Scale bar, 1 μm (**C**) KapD localization in various morphogenetic protein mutant strains. Phase contrast and fluorescence microscopy images at T6 (stages IV–V) of MP and EP mutant cells containing a translational KapD-GFP fusion. In panels A and C an arrowhead indicates the schematized cell shown in the right panel Additional images for panels A, B and D are shown in Supplementary Figure S1. Scale bar, 3 μm. All data shown are representative of at least two independent experiments. (**D**) Western blot of proteins extracted from the coat/crust of density gradient-purified spores from WT, *ΔkapD*, complemented *(ΔkapD/kapD)* and *ΔcotY* mutant strains probed with anti-KapD antibody. Lane M is a molecular weight marker. The 55 kD region of the Coomassie stained gel is shown as a loading control.

Remarkably, the localization of the KapD-GFP protein fusion progressed from a diffuse signal throughout the MC cytoplasm early in sporulation just after asymmetric division, to a single focal point in the mother cell proximal (MCP) forespore pole (closer to the bulk of the MC cytoplasm) at the onset of engulfment when the septal membranes start to curve, then to a cap at both the MCP and the mother cell distal (MCD) poles at completion of engulfment (Figure 3A, Supplementary Figure S2A). Fluorescence was ultimately detected over most of the spore surface, retaining a strong local signal over the MCD polar cap and an apparently decreased intensity at the MCP pole (Figure 3A, phase grey). This behavior is reminiscent of that previously described for class II/III spore coat proteins, which localize to a focal point at the asymmetric septum at the onset of engulfment and encase the forespore after the engulfment process is complete (8), except that a decreased occupation of the MCP pole at later times in sporulation has not previously been observed. Importantly, in control experiments, we have shown that the GFP moiety is not released from the KapD-GFP fusion in mature spores (Supplementary Figure S2C).

To capture KapD localization and dynamics over the spore surface more precisely, we examined the localization of KapD-GFP and TMR-star in dual-color single-cell time-lapse experiments using lattice-SIM (structural illumination microscopy) and the SIM² image reconstruction algorithm. Lattice-SIM² improves optical sectioning and increases resolution 4-fold relative to conventional epifluorescence microscopy (∼60 and ∼250 nm, respectively) (50). The cell-permeable fluorescent dye TMR-star was recently shown to associate specifically with the spore coat and to allow monitoring of the evolution stages of sporulation that cannot be tracked by FM4-64 or by phase contrast (32,51).

Co-localization of KapD and TMR-star throughout sporulation at the single cell level (Figure 3B) confirmed that shortly after completion of engulfment, KapD-GFP transitions from a focal point to a larger cap at the MCP pole of the forespore (Figure 3B, 50 min). A second cap then appears at the MCD pole (Figure 3B, 100 min) and develops into a ‘horseshoe’ pattern (Figure 3B, 150 min), following the disappearance of KapD from the MCP pole (Figure 3B, from 125 min). The horseshoe pattern observed in SIM midplane sections (Figure 3B, 150–225 min) is consistent with midplane epifluorescence images, which contain background fluorescence from the planes above and below due to their limited optical sectioning (Figure 3A, phase grey). Ultimately, by the time TMR-star completely encases the forespore surface, which has been correlated with the completion of inner coat development (32), KapD is only present as a small cap at the MCD pole (Figure 3B, 300 min), and it can still be detected at either the MCD, or at both poles, in fully mature spores (Supplementary Figure S3A). This progression is shown as a short video in Supplementary Movie S1. The production of refractile spores at the end of our single-cell time-lapse experiments confirmed that cells went through the entire sporulation pathway (Figure 3B, Brightfield). Importantly, for later experiments (below), the KapD^AFA^ catalytic mutant displayed the same localization kinetics and pattern as WT KapD (Supplementary Figure S3B).

### KapD locates to the crust layer in a CotY-dependent manner

Morphogenetic proteins play fundamental roles in the building of the spore coat *in vivo* (6). SpoVM and SpoIVA, SafA, CotE and CotZ ensure the construction of the basement, inner coat, outer coat and crust layers of the spore coat, respectively, with CotE being required for both outer coat and crust assembly (8,52,53). The morphogenetic proteins that control assembly of the various coat layers first localize to a focal point at the center of the asymmetric division septum in a SpoIVA- and SpoVM-dependent manner where they recruit the layer-specific proteins. Then, in a second stage controlled by SpoVM and SpoVID (both associated with the basement layer), the layer-specific coat proteins transition from the MCP pole to fully encase the spore (8,54–56). We tested the role of several of these key proteins in KapD localization. In the *safA* mutant, KapD localization was normal (Figure 3C, Supplementary Figure S2B), whereas in the *spoVID* and *cotE* mutants, KapD assembly was either blocked as single foci or polymerized in the MC and failed to spread out to encase the spore. The failure of KapD to assemble correctly in strains lacking CotE narrowed its localization to either the outer coat or crust layer. Remarkably, KapD failed to form foci and remained blocked in the MC cytoplasm in a strain lacking CotY, a homolog of CotZ and a main crust structural component (Figure 3C, Supplementary Figure S2B). The fact that KapD localization on the spore surface is CotY-dependent suggests that KapD is part of the crust layer. Consistent with our observations, the localization dynamics of CotY on the forespore surface is similar to that observed for KapD (32,53), although unlike KapD, CotY completely encases the spore. Localization of a CotY-GFP fusion is independent of KapD (Supplementary Figure S3C), suggesting that it is the recruitment of CotY that brings KapD to the spore crust, rather than a recognition of the CotY–KapD complex.

To confirm the CotY-dependent presence of KapD in the spore coat by an independent method, we performed a Western blot analysis of *B. subtilis* coat proteins extracted from gradient-purified mature spores by boiling in the presence of SDS, β-mercaptoethanol and DTT (57) and probing with an anti-KapD antibody. This experiment showed that KapD was present in the WT spore coat, present at higher levels in a complemented strain expressing KapD from the *thrC* locus, and lacking in both the *ΔkapD* and *ΔcotY* mutants, confirming CotY-dependent localization of KapD to the spore surface (Figure 3D).

### KapD localizes to the MCD pole and is removed from the MC cytoplasm in a SigK-dependent manner

**Figure 4.**
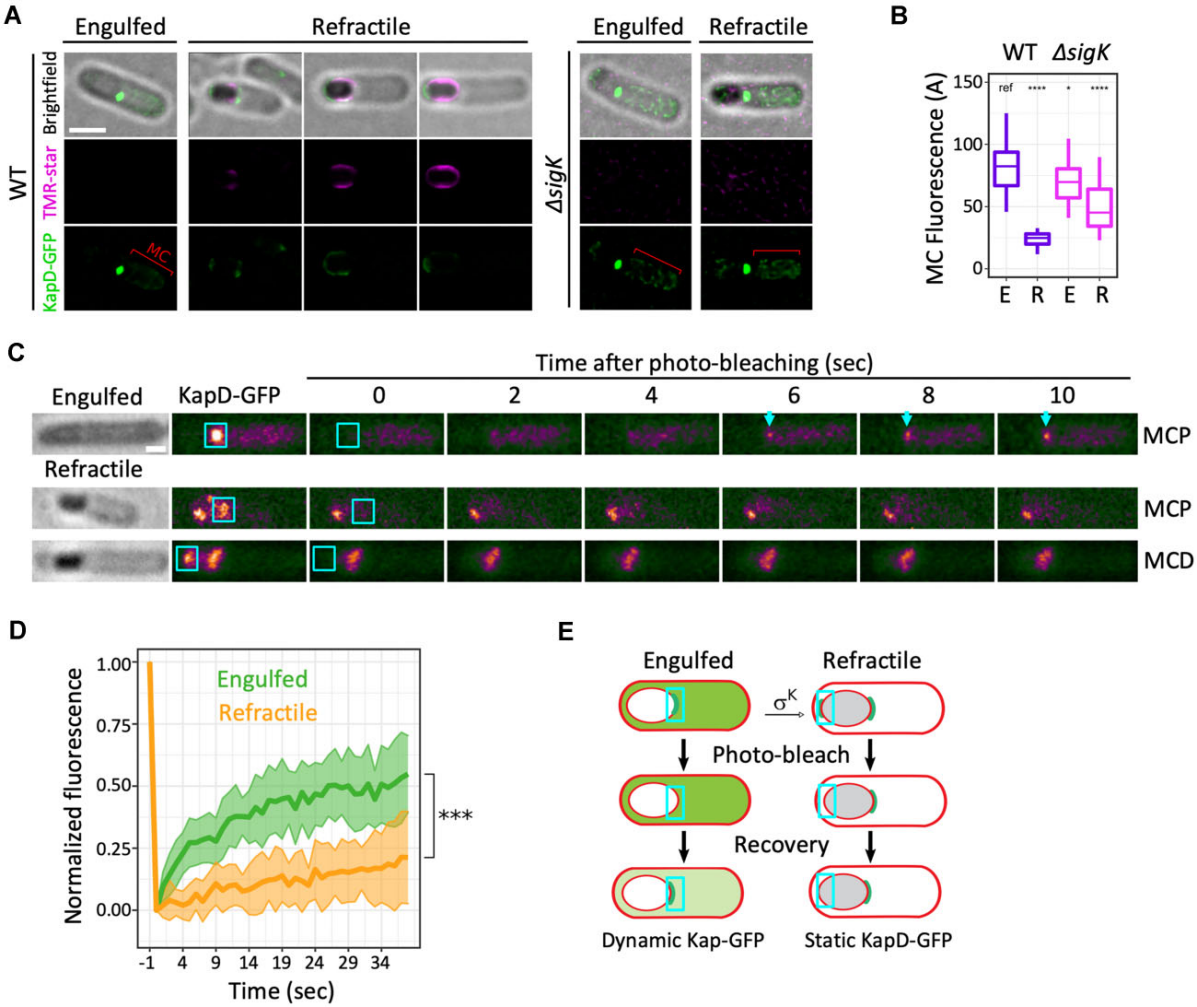
KapD localizes to the MCD pole and is removed from the MC cytoplasm in a SigK-dependent manner. (**A**) Representative SIM^2^ super-resolved localization patterns of KapD-GFP (green) and TMR-star (magenta) in WT (left) and Δ*sigK* (right) cells containing engulfed and refractile forespores, sampled 5.5 and 7 h after onset of sporulation. Localization of KapD-GFP in refractile forespores was classified according to the TMR-star signal. MC, mother cell. Scale bars, 1 μm. (**B**) Quantification of KapD-GFP fluorescent signal intensity in MC containing engulfed (**E**) or refractile (**R**) forespores in the WT (*n* = 26 E; *n* = 31 R) and Δ*sigK* (*n* = 29 E; *n* = 24 R) strains. (**C**) Fluorescence recovery of KapD-GFP in representative engulfed or refractile forespores after photobleaching the ROI indicated by the red square. Scale bar, 0.8 μm. (**D**) Recovery of the fluorescence signal after photobleaching in the ROI in engulfed (*n* = 19) or refractile (*n* = 9) forespores. The average recovery kinetics of bleached cells are indicated by solid lines, and standard deviations are indicated by shaded regions. For quantifications in panels B and D, a non-parametric Mann–Whitney U test was used with the indicated condition as a reference (ref). *, *P*< 0.05; ***, *P*< 0.0005; ****, *P*< 0.00005. (**E**) Schematic representation of FRAP results. In engulfed forespores, fluorescence recovery of the KapD-GFP signal shows a dynamic behavior whereas in refractile spores KapD molecules are static. All data shown are representative of at least two independent experiments.

To better understand to the assembly pathway of KapD into the crust of developing spores and the relative contributions of the two MC-specific sigma-factors, we analyzed the localization of KapD in the *ΔsigK* mutant. In the absence of SigK, the KapD-GFP signal was blocked at the initial focal point formed at the MCP pole at the end of engulfment, indicating that recruitment to the MCD pole is SigK dependent. Since we have shown earlier that SigE is the only other sigma factor responsible for driving KapD expression during sporulation (Figure 2A–D), we conclude that the KapD molecules initially recruited to the MCP pole are primarily synthesized under the control of SigE. The lack of a KapD signal at the MCD pole in *ΔsigK* cells is not due to insufficient protein synthesis, as KapD accumulated in the MC cytoplasm of this strain (at both the end of engulfment and the refractile stage) compared to WT cells where the MC was essentially devoid of KapD by the time forespores became refractile (Figure 4A,B). We conclude that most KapD molecules that localize initially to the MCD pole are also synthesized under the control of SigE, but their removal from the MC cytoplasm and recruitment to this pole requires SigK (perhaps via the synthesis of a recruiting factor). Interestingly, FRAP experiments showed that the KapD-GFP fluorescence signal at the MCP pole of the forespore quickly recovered after photo-bleaching at the end of engulfment, likely representing replenishment through a dynamic exchange with the soluble KapD-GFP sub-population in the MC cytoplasm (Figure 4C–E). However, once spores became refractile, little recovery was observed when the KapD-GFP cap at either the MCD or MCP pole was photo-bleached, indicating that far less exchange occurs at the forespore poles at this point in spore development (Figure 4C–E).

Taken together, these results suggest that early in sporulation KapD is present in two distinct but communicating pools: one at the MCP pole and one in the MC cytoplasm. Later, presumably upon synthesis of one or more SigK-dependent factor(s), all KapD molecules get recruited to the spore surface and the MC is emptied of KapD. The CotY-dependency of KapD localization to the spore surface presented above (Figure 3D) suggested that such a SigK-dependent factor should be present in the crust layer.

### CotY interacts directly with KapD and inhibits its 3′-exoribonuclease activity

**Figure 5.**
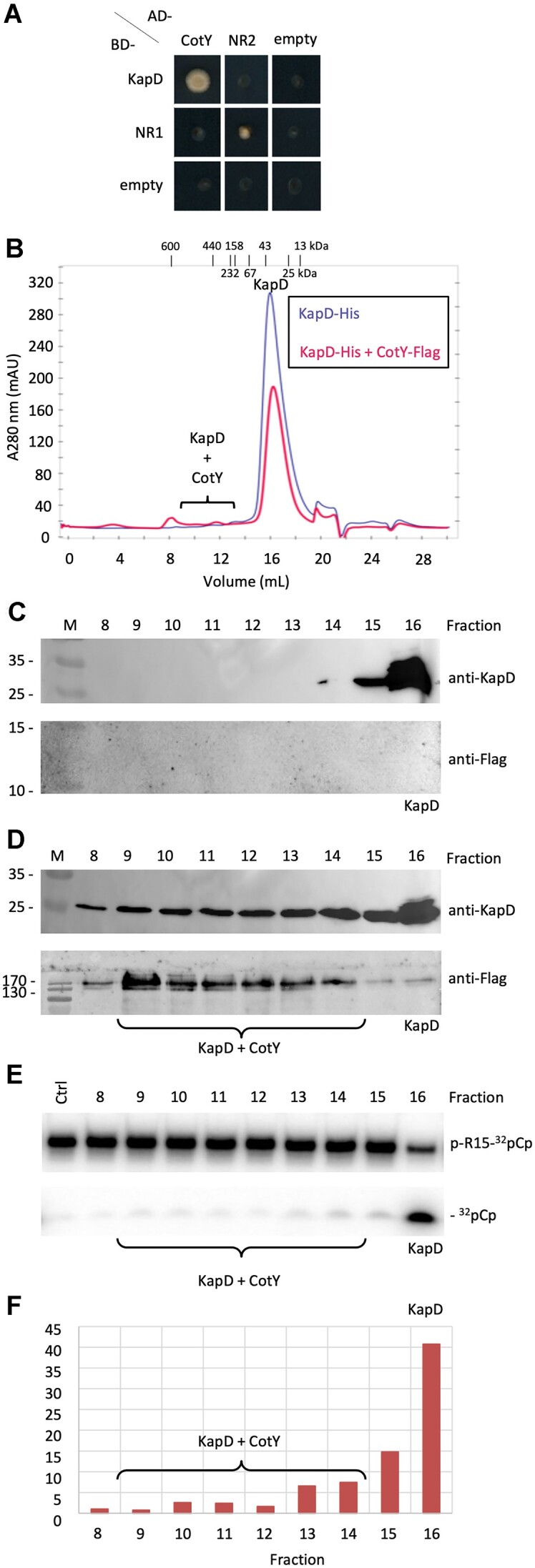
KapD interacts directly with CotY. (**A**) Yeast two-hybrid assay showing a specific interaction between KapD and CotY. Selective plates lack leucine, uracil and adenine (-LUA). AD, Gal4 activation domain; BD, Gal4 DNA binding domain. The AD and BD domains alone (empty vector) and fused to NR proteins, YkpC (NR1) and YhaP (NR2), were used as controls for autoactivation and non-specific activation of the BD-KapD fusion, respectively. (**B**) Gel-filtration profiles of KapD alone (purple) and KapD co-purified with CotY (pink). The migration positions of a molecular weight marker are shown above the chromatogram. (**C**) Western blot analysis of fractions 8–16 from the gel-filtration column of KapD alone probed with an anti-KapD antibody (upper panel) and an anti-Flag antibody (lower panel). (**D**) Western blot analysis of fractions 8–16 from the gel-filtration column of KapD + CotY probed with an anti-KapD antibody (upper panel) and an anti-Flag antibody (lower panel). (**E**) Assay of KapD 3′ exoribonuclease activity in fractions 8–16 from the gel-filtration column of KapD + CotY on ^32^pCp-labeled pR15. (**F**) Specific activity of KapD ± CotY. Results shown are the ratios of the ^32^pCp released (panel E)/KapD amount (panel D), in arbitrary units, for each fraction.

Two lines of evidence suggested that this SigK-dependent factor is CotY itself. First, in a genome-wide yeast two-hybrid screen to search for possible interaction partners of KapD, we identified two independent clones containing the full-length (162 amino acids) CotY sequence. A specificity assay showing growth of one of these clones compared to a strain containing the empty vector or expressing a non-related (NR) protein is shown in Figure 5A. To obtain further evidence in support of this interaction, we constructed an *E. coli* strain producing a C-terminal Flag-tagged derivative of CotY. A pellet of this strain was mixed with a pellet of the *E. coli* strain expressing His-tagged KapD and cells were lysed together before applying the extract to a Ni^2+^-agarose column to purify KapD and any associated CotY. The peak fraction eluted from Ni^2+^-agarose column was then injected onto a gel-filtration column and Western blots were performed on the different fractions using either an anti-KapD antibody or an anti-Flag antibody to identify fractions containing KapD and CotY. KapD alone eluted primarily in fraction 16, consistent with it acting as a 48 kDa homodimer (Figure 5B,C). However, when KapD was co-purified together with CotY, a broad KapD signal (fractions 8–14) of much higher molecular weight (110–600 kDa) was observed in addition to the fraction containing KapD alone (Figure 5B,D). The ability of CotY to self-polymerize (58) likely explains this broad distribution and its migration position on SDS-PAGE. Co-elution of CotY in overlapping fractions (primarily 9–13) was confirmed using the anti-Flag antibody (Figure 5D). A similar profile was observed when the co-purification experiment was performed in the presence of RNase A (Supplementary Figure S4A,B), suggesting that RNA is not required as an intermediate for the KapD–CotY interaction. These experiments, together with our subcellular localization data, provide strong evidence that KapD is recruited to the outermost crust layer of the spore through a direct interaction with CotY, and thus that CotY is a good candidate for the SigK-dependent immobilization of KapD at the MCP and MCD poles late in sporulation described above (Figure 4).

We next tested whether the interaction with CotY had an influence on KapD activity by assaying the 3′-exoribonuclease activity of KapD in the different fractions from the gel filtration column. While KapD alone (fraction 16) showed robust removal of p^32^Cp from the 3′ end of pR15, the fractions containing the KapD–CotY complexes showed very little 3′-exoribonuclease activity (Figure 5E). When normalized for the amount of KapD detected in each lane by Western blot, this experiment showed that CotY inhibits KapD specific activity by 8–10-fold (Figure 5F).

### ***kapD*** mutants have defects in the outer spore coat layers

**Figure 6.**
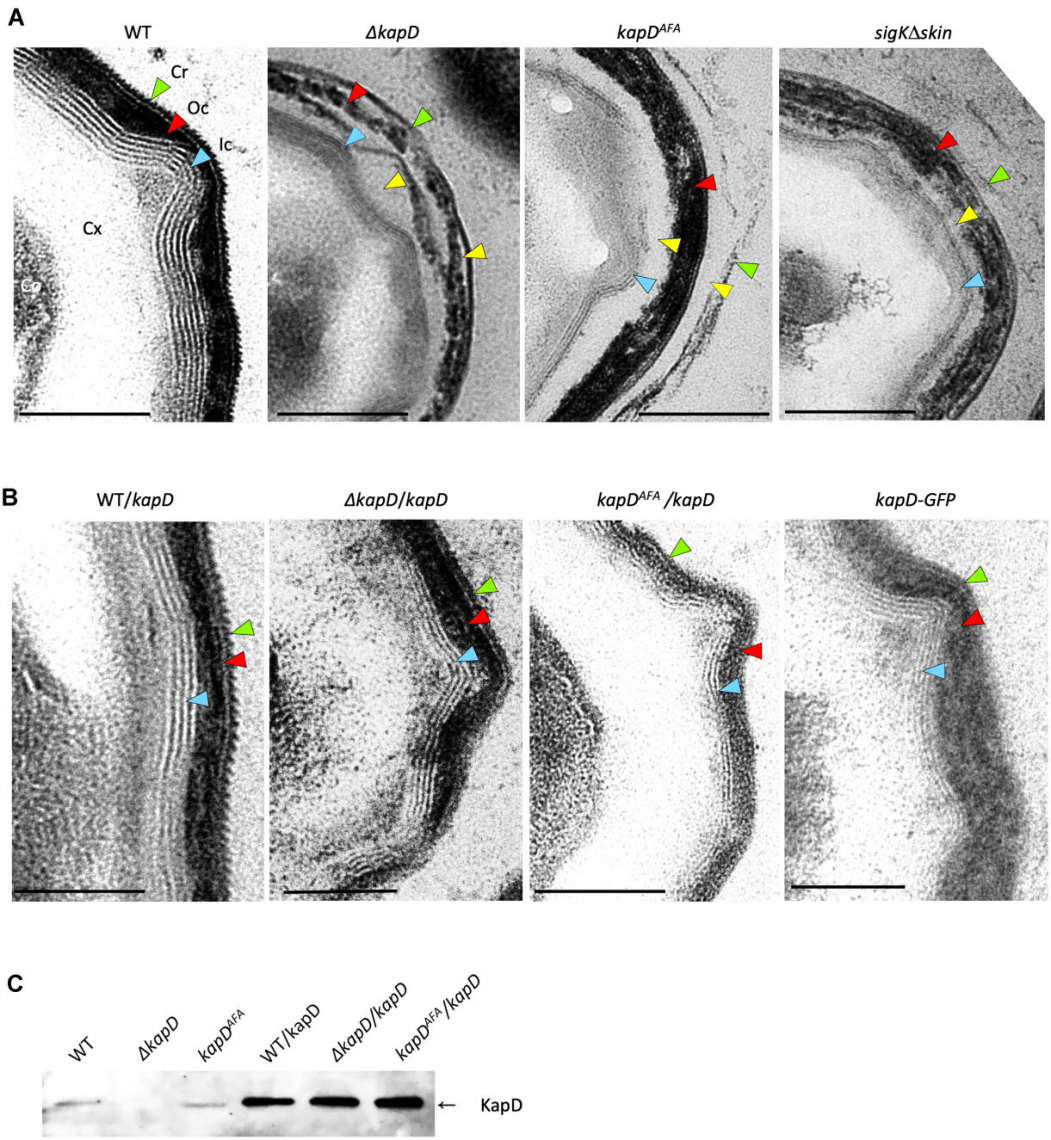
Strains lacking KapD activity have non-cohesive spore coat layers. Representative TEM images of spores isolated from (**A**) WT, *ΔkapD*, *kapD^AFA^*and *Δskin* strains, and (**B**) the WT, *kapD^AFA^* and *ΔkapD*, strains bearing an ectopic copy of the *kapD* gene and the strain expressing the KapD-GFP fusion in place of KapD. The different layers are abbreviated as follows: cortex (Cx), inner coat (Ic; blue arrows), outer coat (Oc; red arrows) and crust (Cr; green arrows). Layer separations are indicated by yellow arrows. Scale bar represents 0.2 μm. (**C**) Western blot showing levels of KapD extracted from purified spores of the indicated strains.

Since our data indicated that KapD localizes to the spore surface, we analyzed the effect of the *kapD* deletion on the ultrastructure of the spore surface by transmission electron microscopy (TEM). TEM images of WT spores showed tightly packed and highly contrasted inner coat, outer coat and crust layers, with few intervening spaces (Figure 6A), as previously reported in similar TEM studies (53,59). In contrast, spores lacking KapD *(ΔkapD*) showed clear defects in the different layers of the spore coat, with poorly defined features, spaces between the inner and outer coats, and between the outer coat and crust layers. Interestingly, the KapD^AFA^ catalytic mutant, present at levels comparable to the WT protein in the spore envelope (Figure 6C), and that assembles with a similar pattern and kinetics to the WT protein (Supplementary Figure S3), showed a similar highly disrupted structure of the multilayered coat and crust (Figure 6A). Thus, KapD RNase activity is not important for KapD localization on the spore surface, but is essential for spore coat/crust integrity. Interestingly, we saw a similar disrupted layer phenotype for spores from a strain lacking the *skin* element (Figure 6A, last panel). The significance of this observation will be discussed below. Complementation of the *ΔkapD* and *kapD^AFA^* catalytic mutant strains with an ectopic copy of the *kapD* gene that produces an excess of KapD compared to expression from the native locus (Figure 6C), allowed recovery of the WT pattern, confirming that the phenotypes described above are indeed caused by the lack of KapD activity (Figure 6B). The strain expressing the KapD-GFP translational fusion from the native *kapD* locus also had a WT layer pattern, confirming that this fusion is fully functional (Figure 6B, last panel). Despite the defect in the integrity of the spore coat layers in the *ΔkapD* strain, the spores have similar heat resistance and germination rates to WT spores (Supplementary Table S4).

### The absence of KapD has a strong impact on spore coat protein levels

Since mutations in *kapD* lead to a highly disrupted spore coat/crust morphology, we examined the protein composition of these outer layers to ask whether their levels changed in a KapD-dependent manner. We extracted spore-coat proteins from WT and *ΔkapD* spores and analyzed them by mass-spectrometry (two aliquots of five biological repeats for each strain). Although about 1400 different proteins were identified in this analysis (Supplementary Table S1), we focused our attention on about 280 of the most abundant proteins known to be part of the spore-coat or members of the SigE and SigK regulons, and therefore likely to be co-expressed with KapD. We identified 20 upregulated proteins and 25 down-regulated proteins that had a student test *P*-value < 0.05, or were detected in at least three biological replicates in one strain (Table 1; Supplementary Figure S5A). Of the upregulated proteins, only three were known spore coat/crust components (CotW, present in the outer crust layer, YhjR of unknown function and SpoIVA, a morphogenetic protein involved in basement layer assembly). However, the mRNA levels for these three proteins did not vary significantly in a *ΔkapD* strain compared to WT during the 8 h of spore development, suggesting that they are not substrates of KapD exoribonuclease activity (Supplementary Figure S5B,C) and that the effects seen in the proteomic data are likely at the translational or post-translational or assembly levels, and thus indirect.

**Table 1.** Mass spectrometry analysis of spore coat proteins^1^

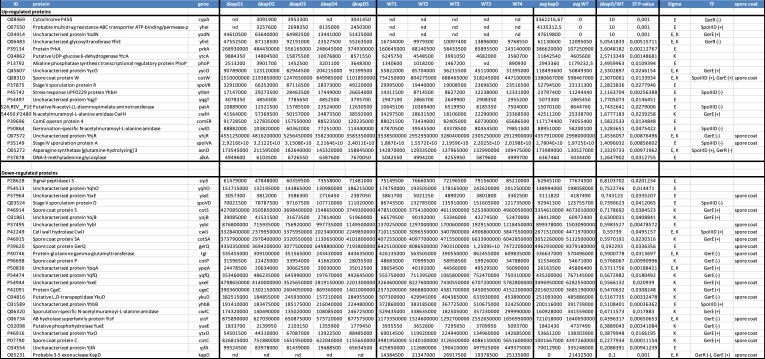

^1^Proteins with a student T-test (ST) *P*-value of < 0.05 or present in at least three samples (*P*-value = 1) are shown. nd is not detected. TF is transcription factor. Note that candidates not detected in WT or ΔkapD strains were arbitrarily attributed a fold-change of 10 and a *P*-value o 0 001.

Fourteen of the 25 downregulated proteins were known spore-coat proteins, 17 were members of the SigK regulon and 13 were controlled by SigE (5 were members of both regulons). While the downregulation of multiple spore coat proteins could potentially explain the adhesiveness defect observed in the *kapD* mutant spore-coat layers, it was surprising that a lack of a 3′-exoribonuclease, which would be predicted to primarily increase mRNA stability, would lead to more down-effects on spore coat protein levels than up-effects. We therefore surmised that these effects were also likely to be indirect via a common effector.

### KapD controls the stability of the sigK mRNA

**Figure 7.**
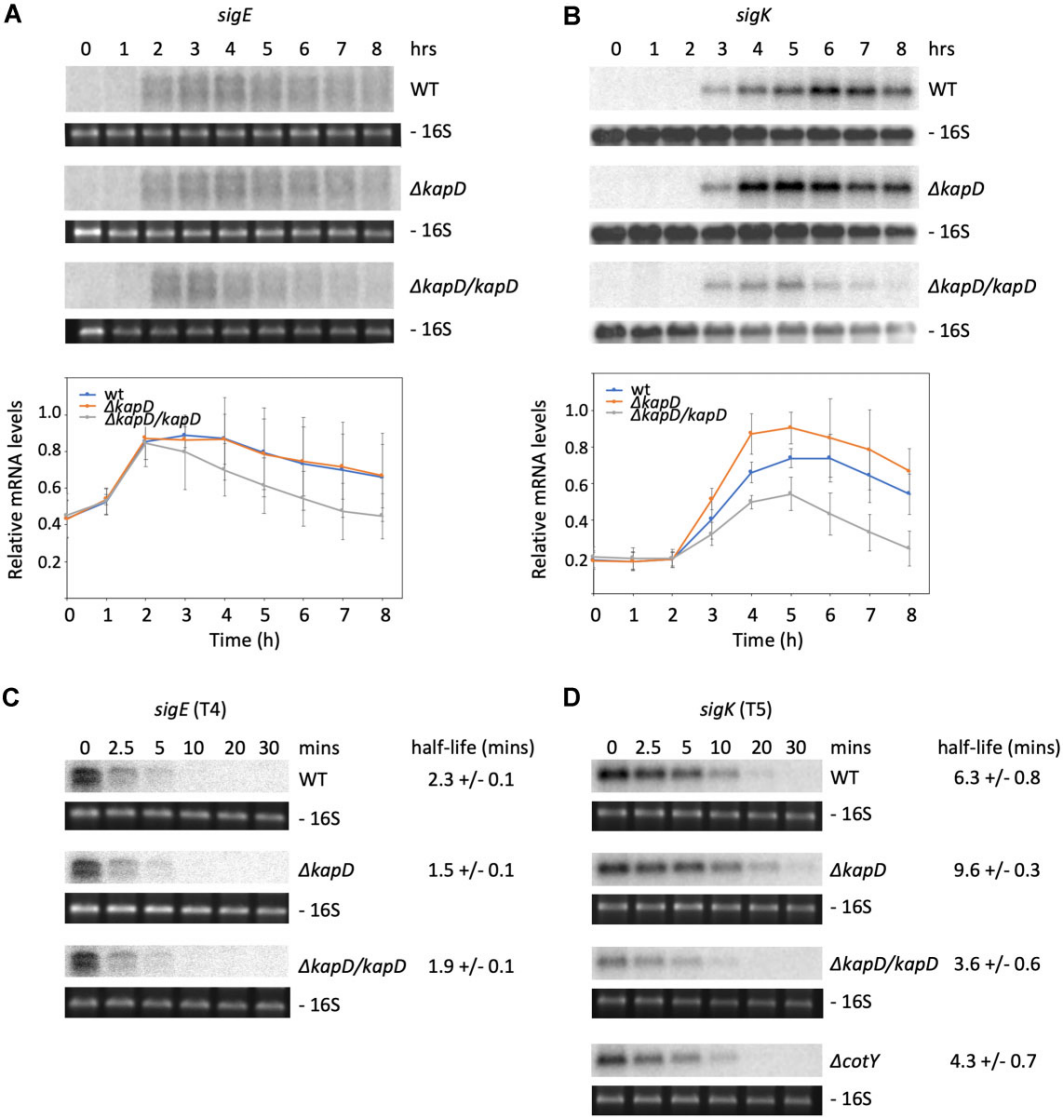
Strains lacking KapD have increased and early *sigK* mRNA expression, correlated with increased *sigK* mRNA stability. Northern blot showing expression of (**A**) *sigE* and (**B**) *sigK* (post-skin exision) in total RNA isolated from WT, *ΔkapD* and complemented (*ΔkapD/kapD*) cells 0–8 h after resuspension in sporulation medium, probed with oligos CC2760 and CC2765, respectively. Ethidium bromide stained or 16S probed (CC058) rRNA is shown under each gel as a loading control. A quantification of three independent experiments is shown below each autoradiogram. Signals were normalized to the maximal value for each membrane containing WT, *ΔkapD* and *ΔkapD/kapD* samples. Error bars represent standard deviation. Northern blot showing expression of (**C**) *sigE* and (**D**) *sigK* mRNAs at times after rifampicin addition to WT, *ΔkapD* complemented (*ΔkapD/kapD*) or *ΔcotY* cells 4 (T4) and 5 (T5) h, respectively, after resuspension in sporulation medium. Probes were the same as in panel A. Half-lives were calculated from two independent experiments, represented graphically in Supplementary Figure S6.

Since the downregulated proteins in the proteomics experiment were all members of the SigE and SigK regulons, we looked specifically at the profiles of these two mRNAs throughout sporulation in the *ΔkapD* mutant and in the strain overexpressing KapD. Although *sigE* mRNA levels were decreased at later time points in the strain that overexpresses KapD, the expression profile did not vary significantly between the WT and *ΔkapD* mutant (Figure 7A). However, a clear impact was observed for the *sigK* mRNA in the absence of KapD, where expression was reproducibly stronger at earlier time points (T4/T5) compared to the WT strain, which had a peak around T5/T6 (Figure 7B). In the complemented/overexpressing strain (*ΔkapD/kapD)*, *sigK* expression was strongly reduced at every time point (Figure 7B). These results showed that KapD negatively affects the levels of the *sigK* mRNA.

We next asked whether the impact of KapD on *sigK* mRNA levels was direct, *i.e*. at the post-transcriptional level. While deletion or overexpression of *kapD* had no effect on the half-life of the *sigE* mRNA (measured after rifampicin addition to cultures to block transcription), deletion of *kapD* lead to a 1.5-fold stabilization of the *sigK* mRNA (9.6 versus 6.3 min half-life in *ΔkapD* and WT strains, respectively) (Figure 7C,D; Supplementary Figure S6A,B). The corollary was also true: overexpression of KapD in the complemented strain reduced the *sigK* mRNA half-life 1.8-fold, to 3.6 min. We conclude that the *sigK* mRNA is a *bona fide* substrate of KapD.

### Sequestration of KapD in the spore crust is a means of reducing its activity in the MC

Finally, we asked why might it be beneficial for *B. subtilis* to incorporate the KapD RNase into the spore crust. Our fluorescence microscopy data (Figure 3C, Supplementary Figure S2B) suggested that sequestration in the spore crust via direct protein-protein interaction with CotY is a way of reducing KapD exoribonuclease activity in the MC. To test whether this was relevant to *sigK* expression, we measured the stability of the *sigK* mRNA in the Δ*cotY* mutant, where KapD remains in the MC (Figure 3C). In the absence of CotY, the half-life of the *sigK* mRNA (4.3 min) was significantly decreased compared to WT (6.3 min) (Figure 7D, Supplementary Figure S6B) and similar to that observed in the KapD complementated strain (3.6 min). This suggests that at least one reason for the CotY-dependent spore crust localization of KapD is indeed to remove it from the MC cytoplasm and inhibit its activity, thereby relieving inhibition of *sigK* expression.

## Discussion

Our findings reveal that the *sigK* mRNA is a major substrate for the sporulation-specific KapD 3′-exoribonuclease in *B. subtilis*. In the *ΔkapD* strain, *sigK* mRNA expression occurs earlier and at higher levels compared to WT, whereas in the complemented strain overexpressing KapD, *sigK* mRNA levels are markedly reduced. This correlates with an effect of KapD on *sigK* mRNA stability, indicating that the effect is at a post-transcriptional level and that this mRNA is a true substrate of KapD. Thus, we provide evidence for a fourth level of control of SigK expression/activity and show it is first controlled at a post-transcriptional level by KapD and then counteracted at late stages by CotY-dependent recruitment to the crust. The existence of multiple, intricate SigK regulatory systems underscores the critical importance of precisely controlling this mother-cell-specific sigma factor for correct spore development. RNA sequencing experiments in the *kapD* deletion mutant at different times during sporulation, followed by Northern blotting of potential differentially expressed candidates, failed to find any additional direct substrates of KapD (unpublished data). The overexpression of KapD in the complemented strain *(ΔkapD/kapD*) resulted in down-regulation of many mRNAs tested by Northern blot. However, most of these appear to be due to indirect transcriptional effects (perhaps linked to the downregulation of SigK), as their half-lives were unaltered (data not shown). Thus, for now, KapD seems to be a relatively specific post-transcriptional regulator of the *sigK* mRNA.

KapD is the first 3′-exoribonuclease known to be sensitive to the phosphorylation status of the 5′ end of its mRNA substrates. Stimulation of KapD activity by a 5′-monophosphate (5′-P) compared to a 5′-hydroxyl (5′-OH) group or a 5′-triphosphate suggests that KapD may play a role in the degradation of RNA intermediates that have been produced by other endoribonucleases such as RNase Y or RNase III, which liberate 5′-P-containing fragments, rather than those generated by toxin RNases, which typically yield 5′-OH ends, or primary transcripts. The end-products of KapD digestion of the 5′-labeled substrate were 9–11 nts, suggesting that it loses processivity when it has trimmed its RNA substrates down to this size. By comparison, the 3′-exoribonucleases RNase II and RNase R of *E. coli* lose processivity in the 4–5 and 2–4 nt range, respectively (60–62). This can be rationalized in terms of the nt distance between the catalytic site and the closest RNA binding site on the protein, and suggests that this distance is about 9–11 nts in the case of KapD.

DEDDh-family 3′-exoribonucleases are generally single-strand specific RNases, whose progress is inhibited by secondary structure (63). The best-known examples in bacteria are oligoribonuclease (Orn), required for the degradation of very short RNAs (2–6 nts) (64), and RNase T, involved in rRNA and tRNA 3′-maturation (63). In addition to being blocked by secondary structure, RNase T is also strongly inhibited by C-residues that prevents it from degrading past the two C’s of the CCA motif required for aminoacylation (65). The eukaryotic DEDDh enzymes 3′hExo and Eri1 additionally possess an SAP (SAF-box, Acinus and PIAS) domain important for their recruitment to RNAs with double-stranded regions, including the stem-loop of the histone mRNA, and si- and mi-RNAs, respectively (66). Recruitment to RNA duplexes improves the efficiency of the 3′-trimming reaction of the single-stranded portion of these RNAs.

It is unusual for mRNAs to be sensitive to 3′-exoribonucleases as the hairpin structure of intrinsic terminators is usually strong enough to prevent exoribonuclease access. The average thermostability of terminator hairpins in *B. subtilis*, measured as Gibbs free energy (ΔG), is −16 kcal per mol and they are typically followed by a run of 5–7 U-residues (67). Although there is a stem loop just downstream of the *sigK* coding sequence, its ΔG is −9 kcal/mol and it is followed by only 3 consecutive U-residues. Furthermore, there is evidence of increased transcriptional signal downstream of the *sigK* coding sequence in the absence of termination factor Rho (13), suggesting that *sigK* may be primarily subjected to Rho-dependent termination. Rho termination sites are known to be particularly vulnerable to 3′-exoribonuclease attack as they are typically single stranded. This, combined with the lack of a strong hairpin downstream of the coding sequence may explain the particular sensitivity of the *sigK* mRNA to KapD. The mammalian homolog of KapD, 3′hExo, employs a stem-loop binding protein (SLBP) to prevent it from prematurely degrading histone mRNAs during the cell cycle (68). Although *B. subtilis* has no homolog of SLBP, we cannot rule out that KapD activity is regulated by a protein(s) with functionally similar properties that could account for its apparent specificity for the *sigK* mRNA.

Although Bacillaceae and Clostridiales share a globally conserved pathway to sporulation, there are some significant differences (e.g. SigK does not have an inhibitory pro-sequence in Clostridiales) (69). Of the two spore-forming groups, KapD is specific to the Bacillaceae, including several important human or insect pathogens such as *B. anthracis*, *B. cereus* and *B. thuringiensis*. It should be noted that KapD is also conserved in some Bacillaceae, such as *B. cereus*, that lack the *skin* element and associated recombinase (51). Thus while *B. subtilis* has the luxury of four levels of control of SigK expression or activity, several spore-formers may only have two or three. Homologs of KapD are also scattered among some non-spore formers, including some species of *Staphylococcus*, *Streptococcus*, and even in some Proteobacteria such as *Vibrio*, *Pseudomonas* and in certain strains of *E. coli*. KapD has presumably been acquired by horizontal gene transfer in these organisms, in which its function remains unknown.

Despite the increased *sigK* expression in the *ΔkapD* mutant, proteomic analysis of the outer spore layers revealed that many members of the SigK regulon were actually present at lower levels compared to WT. While the decrease in some combination of the 14 spore-coat proteins could account for defects observed in the outer spore layers by TEM, none are known to produce a similar phenotype when individually absent. Mutations that have previously been associated with defective adhesion of the spore coat layers include deletions of the *safA* (70), *spoVID* (55), *ywcE* (71), *cotG* (57), *sodA* (59) and *rho* (72) genes. We checked the levels of each of the encoded proteins in the proteomics data. SafA, SpoVID and SodA did not show significant variation, YwcE and CotG were not detected and Rho was increased 2-fold in the *ΔkapD* strain, *i.e* opposite to the effect of the *rho* deletion that had a defective layer phenotype (72). Since *safA* and *spoVID* encode key morphogenetic and encasement proteins for the inner coat assembly and encasement respectively, we also analyzed their mRNAs levels during the sporulation cycle. Consistent with the proteomics data, neither mRNA varied significantly in the Δ*kapD* mutant compared to WT (Supplementary Figure S7).

The reduced levels of many spore coat proteins in the Δ*kapD* mutant suggests a deficiency in SigK activity, yet paradoxically the lack of KapD stabilizes the *sigK* mRNA. If decreased synthesis (or assembly) of these proteins is responsible for the spore coat phenotype observed in the *ΔkapD* mutant, this would suggest that early and/or higher levels of *sigK* expression can also perturb the balance of the different components responsible for the adhesion of outer spore layers, i.e. that too much/too early SigK may be similarly deleterious to expression that is too little/too late. Consistent with this idea, perturbing the pro-SigK to SigK checkpoint in *B. subtilis* affects the functional properties of the spore (22) and, as we show here, removal of the *skin* element also reduces adhesiveness of the coat sub-layers (Figure 6A). Deletion of the *skin* element in *Clostridioides difficile* also leads to premature expression of *sigK* and to an altered spore morphology (73), resonant with the *ΔkapD* phenotype seen by TEM (Figure 6A). Furthermore, a *ΔcotY* mutant, in which KapD remains fully in the MC, also shows gaps between the spore coat layers (53). We have shown that in this context the *sigK* mRNA is unstable. The fact that there is evidence of a similar layer phenotype both in the absence or at higher levels of KapD is coherent with the idea that deviation in either direction from an optimal amount or timing of *sigK* expression can perturb the adhesiveness of the outer spore layers.

Our data reveal a curious and totally unexpected localization pattern for a ribonuclease. Although some ribonucleases (e.g. RNase I, (74)) can be localized in the periplasmic space or are secreted outside of vegetative cells (e.g. YurI (75)), most carry out their functions within the cell, either in the cytoplasm (e.g. RNase III (76)) or associated with the inner membrane (e.g. RNase E and RNase Y (77,78)). KapD starts out as a soluble protein in the cytoplasm MC, where its role seems to be primarily to dampen *sigK* mRNA levels and prevent premature expression. Then, as the engulfment process proceeds, KapD first gets recruited to a restricted zone at the MCP pole, presumably through its direct interaction with a pool of CotY molecules that are also synthesized initially under control of SigE. KapD localization then expands to an MCP cap and, upon completion of engulfment, a second cap at the MCD pole, under SigK-control. However, unlike most spore coat proteins with a similar behavior (8), KapD never fully encases the spore. Instead, it is first removed from the MCP pole, and then from the long sides of the spore before eventually retracting to a small cap at the MCD pole (Figure 3B, Supplementary Figure S3A). The mechanism underlying this dynamic distribution of KapD remains unknown and has not been previously described for a coat protein. Nonetheless, our data show that its integration into the spore crust through its interaction with CotY plays an important physiological role in linking the different stages in the morphology of the spore surface layers to the expression of the key developmental sigma factor SigK.

**Figure 8.**
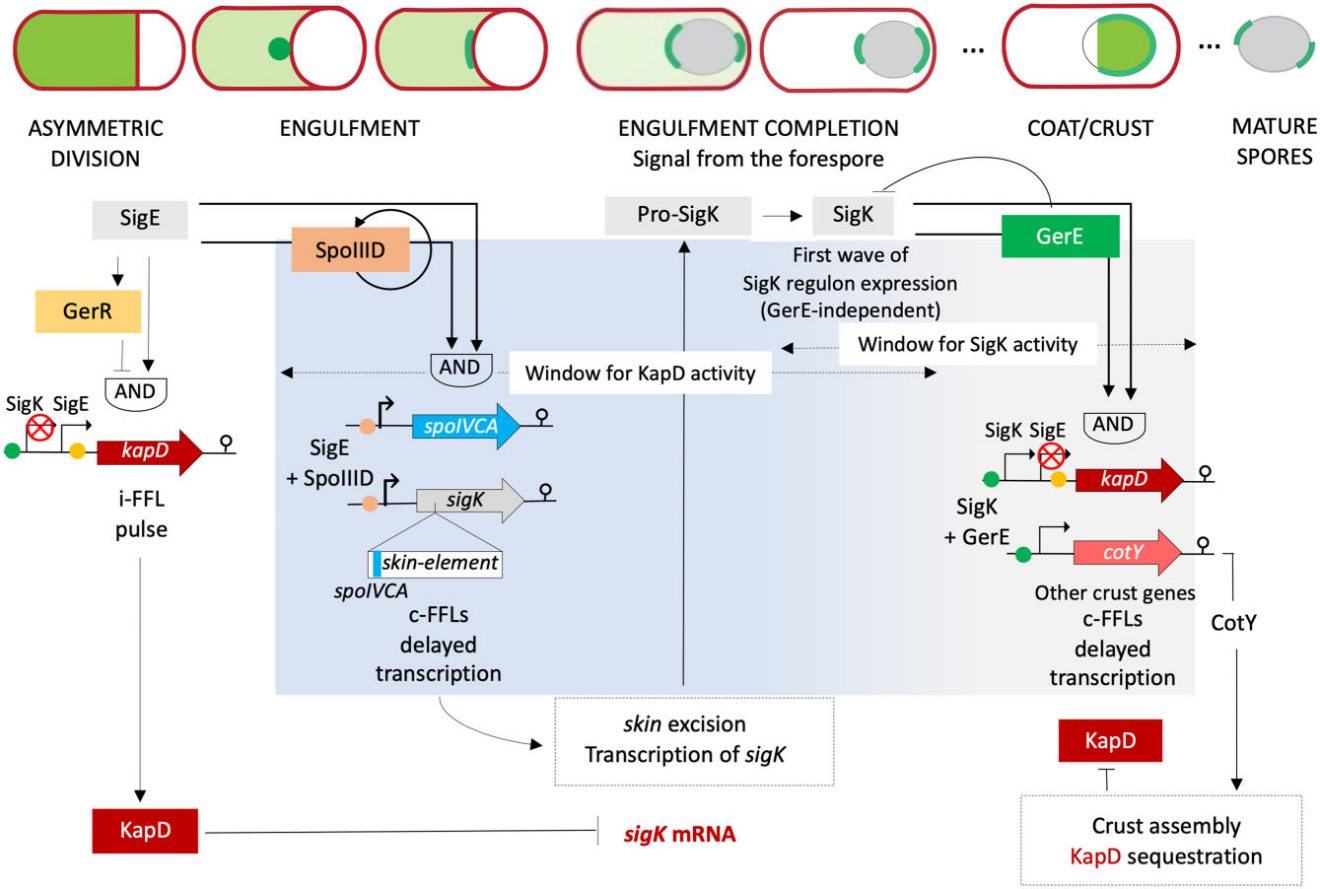
Model of transcriptional and post-transcriptional regulation of *sigK*. The top row depicts the relevant morphological stages of sporulation and the pattern of expression and sub-cellular localization of a KapD-GFP fusion. The model highlights the coherent (c) or incoherent (i) feed forwarded loops that create a pulse of *kapD* transcription or delayed transcription of *kapD* and other relevant genes. Note that the transcription of both *sigK* and of *spoIVCA* (coding for the recombinase involved in excision of the *skin* element) is delayed relative to the transcription of other SigE-dependent genes and takes place toward the end of the engulfment sequence. The dots represent binding sites for the ancillary transcription factors represented, GerR, SpoIIID and GerE and the red X, the switch from the SigE (to the SigK-dependent *kapD* promoters. When SigK becomes active following engulfment completion, both GerE (at the transcriptional level) and possibly KapD (at the post-transcriptional level) negatively regulate *sigK* expression. Early expression of *kapD* under SigE control modulates a first wave of SigK regulon expression, prior to a second, GerE-dependent wave. Both CotY and KapD are then expressed under the control of GerE, and KapD is recruited to the crust by CotY. The burst in *kapD* transcription at this stage may indicate that KapD has an additional role at the spore surface. For a full description of the model, see text.

The model shown in Figure 8 summarizes our current understanding of SigK regulation at the transcriptional and post-transcriptional levels and emphasizes how the redundancy resulting from multilevel control is important for correct morphogenesis. Early in sporulation, prior to engulfment completion, *kapD* expression occurs as a pulse (see Figure 2D), due to an incoherent feed-forward loop established by SigE, which drives *kapD* transcription, and by GerR, also a member of the SigE regulon, which represses it (12). Both the excision of the *skin* element and transcription of the *sigK* are delayed toward the end of the engulfment process. This is because transcription of the gene coding for the recombinase SpoIVCA and transcription of *sigK* both require SigE and the auto-regulatory SpoIIID accessory factor, which define a coherent feed-forward loop that delays transcription until SpoIIID accumulates (12,20,79). The genomic rearrangement that produces a functional *sigK* gene is thought to occur before transcription of the reconstituted gene. Finally, SigK is synthesized as an inactive form (pro-SigK) and is activated by a protease cascade initiated in the forespore concomitant with engulfment completion and involving multiple factors (22,80,81). These factors have been omitted from Figure 8 for simplicity. These mechanisms, collectively termed the SigK checkpoint, effectively couple the onset of SigK activity to engulfment completion. Our data suggests that early expression of KapD under control of SigE contributes to the SigK checkpoint in that it may inhibit inappropriate appearance of the *sigK* mRNA prior to engulfment completion and that this is important for correct spore formation. In line with this idea, it has been shown that perturbing redundancy and robustness of the SigK checkpoint allows inappropriate activity of SigK under non-sporulation conditions (19). Once SigK is activated, it takes over the control of *kapD* transcription, together with GerE, also under SigK control. Transcription of *cotY* is also under the joint control of SigK and GerE. The resulting coherent feed-forward loops delay maximal activation of both *kapD* and *cotY* transcription (12). GerE then inhibits *sigK* transcription. Thus, SigK is responsible for the synthesis of two inhibitors of its own mRNA levels: GerE, at the transcriptional level and KapD at the post-transcriptional level. At later stages, KapD is removed from the MC cytoplasm and associates with the crust through its interaction with CotY (Figure 5), such that it can no longer impact *sigK* expression. Inhibition of KapD activity through its interaction with CotY may allow for a brief increase in SigK expression before it is transcriptionally shut down by GerE. The fact that KapD continues to accumulate and is detected near the poles of mature spores despite its activity being inhibited by CotY, suggests that it may have an additional role in spore coat/crust assembly that remains to be discovered.

## Supplementary Material

gkae1301_Supplemental_Files

## Data Availability

The raw proteomics data in Supplementary Table S1 have been deposited at ProteomeXchange, accession number PXD058725.
