# Supplementary material for: Embedding a ribonuclease in the spore crust couples gene expression to spore development in Bacillus subtilis
Source: Nucleic Acids Res. 2025 Jan 16;53(2):gkae1301. doi: 10.1093/nar/gkae1301 (PMC11736430; doi:10.1093/nar/gkae1301)
Supplement: gkae1301_Supplemental_Files [file gkae1301_supplemental_files.zip › 2024 KapD NAR Supp Tables and Figures revised.pdf]

Table S2: Strains used for this study

	Genotype	Reference
B. subtilis		
SSB1002	W168 *trpC+*	Lab strain
CCB069	W168 *Pspac-kapD ery*	This study
CCB320	W168 *sigE::ery*	This study
CCB496	W168 *sigK::tet*	This study
CCB566	W168 $kapD^{RFR}$	This study
CCB567	W168 *kapD::ery*	This study
CCB607	W168 *kapD::spc-eryTer*	This study
CCB786	W168 *KapD-GFP-pCVO119 spc* (translational fusion)	This study
CCB861	*W168 KapD-GFP-pCVO119 sigK::tet*	This study
CCB902	W168 *KapD-GFP-pCVO119 spoIIQ::ery*	This study
CCB904	W168 *KapD-GFP-pCVO119 spoVID::ery*	This study
CCB906	W168 *KapD-GFP-pCVO119 cotY::ery*	This study
CCB908	W168 *KapD-GFP-pCVO119 cotE::ery*	This study
CCB909	W168 *KapD-GFP-pCVO119 safA::ery*	This study
CCB1251	W168 $kapD^{AFA}$	This study
CCB1372	*W168* $kapD^{AFA}$*-GFP-pCVO119*	This study
CCB1373	W168 *thrC::pDG1664-kapD*	This study
CCB1374	W168 *thrC::pDG1664-kapD* $kapD^{AFA}$	This study
CCB1375	W168 *thrC::pDG1664-kapD kapD::spc-eryTer*	This study
CCB1744	W168 *thrC::pDG1664-rrnBt1t2-kapD*	This study
PY79	Parental strain	Youngman et al (1)
PY786	PY79 *KapD-GFP-pCVO119*	This study
PE3158	PY79 *amyE::cotY-gfp cm*	Shuster et al (2)
PE3158Δ	PY79 *amyE::cotY-gfp cm kapD::ery*	This study
E. coli		
CCE095	BL21C+ *pET28a-KapD-His kan (pRIL: argU ileY leuW cm)*	This study
CCE273	BL21C+ *pET28a-*$KapD^{AFA}$*-His kan (pRIL: argU ileY leuW cm)*	This study
CCE275	BL21C+ *pET28a-CotY-3xFlag kan (pRIL: argU ileY leuW cm)*	This study

1. Youngman, P., Perkins, J.B. and Losick, R. (1984) Construction of a cloning site near one end of Tn917 into which foreign DNA may be inserted without affecting transposition in Bacillus subtilis or expression of the transposon-borne erm gene. *Plasmid*, **12**, 1-9.
2. Shuster, B., Khemmani, M., Abe, K., Huang, X., Nakaya, Y., Maryn, N., Buttar, S., Gonzalez, A.N., Driks, A., Sato, T. *et al.* (2019) Contributions of crust proteins to spore surface properties in Bacillus subtilis. *Mol Microbiol*, **111**, 825-843.

Table S3: Oligonucleotide used for this study

Oligo	*Gene*	Sequence 5'-3' (non-hybridising sequences in lower case)
CCR018	-	UGGUGGUGGAUCCCG
CCR034	-	AGGUGGUGGAUCCCG
CCR036	-	GGGUGGUGGAUCCCG
CC073	*kapD*	ATATGAATTCGAAAGGAGGTGTAAAAGGTGACGAC
CC074	*kapD*	ATATAGTCGACCGTCGCGCGCTTCAACAGTTC
CC890	*kapD*	CTTTCCATAAACCCGTCAATGTCCGGTCTC
CC1010	*kapD*	GGTTTTAGTCCACTCTCAACTCCTGATC
CC1196	*kapD*	CAAACAGCTTACTCATTATTCGTTTCCGATTTACAATGCCTGATGG
CC1197	*kapD*	CCATCAGGCATTGTAAATCGGAAACGAATAATGAGTAAGCTGTTTG
CC1354	*kapD*	GACAGGTCCGTGCAGCTCATTTAC
CC1355	*kapD*	CGGAGGTGTAGCATGTCTCATTCcaccttttacacctgcctttcctccg
CC1356	*kapD*	GGCTTAAACCAGTTTTCGCTGGTGCGaccgaactgttgaagcgcgcgacg
CC1357	*kapD*	GCTTGTACTGTCAGAGCTTGACCG
CC1365	*ery*	cggaggaaaggcaggtgtaaaaggtgGAATGAGACATGCTACACCTCCG
CC1366	*ery*	cgtcgcgcgcttcaacagttcggtCGCACCAGCGAAAACTGGTTTAAGCC
CC1370	*kapD*	GCGAGCAGGGGCTGGATACTGAAATTG
CC1371	*kapD*	CATTATCACAAACTATCTCGATATGGCC
CC1375	*kapD*	atatgtcgacGTACACCGATGTAACAAAGCCCATG
CC1376	*kapD*	atatgaattcGCTGTGAGAGCATCATCAAGCGC
CC1436	*kapD*	CGAACGAAAATCGCCATTCGCCAGcaccttttacacctgcctttcctccg
CC1437	*spc*	cggaggaaaggcaggtgtaaaaggtgCTGGCGAATGGCGATTTTCGTTCG
CC1438	*spc*	GCAGTTTATGCATCCCTTAACTTACTATGCAAGGGTTTATTGTTTTCTAAAATC
CC1439	*ery*	GATTTTAGAAAACAATAAACCCTTGCATAGTAAGTTAAGGGATGCATAAACTGC
CC1457	*kapD*	TAAGTTAAGGGATGCATAAACTGC
CC1458	*kapD*	GACGAAACCCTACAAAACAAGTCGAGTGCG
CC1468	*kapD*	ccg agc ccc gtt gct gaa aat gcc
CC1837	*yhjR*	GTGGGCGAGTTCGGCAAGCTTGCGGTAGC
CC2054	*kapD*	AGCTTatcggtGACTACAAGGACCACGACGGTGACTACAAGGACCACGACATCGACTA CAAGGACGACGACGACAAGTGAc
CC2055	*kapD*	TCGAgTCACTTGTCGTCGTCGTCCTTGTAGTCGATGTCGTGGTCCTTGTAGTCACCGT CGTGGTCCTTGTAGTCaccgatA
CC2058	*kapD*	ATATGAATTCagtcaaataccctataaagaaggagctg

CC2059	*kapD*	ATTAAAGCTTtccattgtgatgatgctttttatctttgtg
CC2070	*kapD*	caaacagcttactcattattgctTTCGCATTTACAATGCCTGATGG
CC2071	*kapD*	CCATCAGGCATTGTAAATGCGAAagcaataatgagtaagctgtttg
CC2524	-	ATTAATACGACTCACTATAGTGGTGGTGGATCCCG
CC2525	-	CGGGATCCACCACCACTATAGTGAGTCGTATTAAT
CC2580	*cotB*	CCCTGCCCCAAGTCGCATCATAATCCC
CC2588	*sigK /sigE*	ccgctagcacaacgatgatcgCTCACAGTGGGAGAAATGGC
CC2589	*sigK /sigE*	GCCATTTCTCCCACTGTGAGcgatcatcgttgtgctagcgg
CC2590	*kapD*	atatgaattcaaaaaccggtaggactgcctaccggTTAGCTCCCTTCATAATCTGGAC
CC2605	*sigK*	atatgaattcGACGTAGGAAACGTAGTCAGG
CC2674	*cotW*	GTTCTTTTGCTCTTGTGCGGCTGCTTCTTC
CC2681	*spoIVA*	GGCCGACAACCCTGTCTACGTCCCGGAGCCG
CC2682	*safA*	CATCGGCATATTCGGAACTGTGTGATGAACG
CC2689	*spoVID*	GGCTGGCGGTTCATGCTGAAGAACTGCTTCC
CC2760	*sigE*	GCCGCCTGATCGCCGTTTGGGAGCTTC
CC2765	*sigK*	CCCGACAATCACTTCCTTCTCACGGTCATC
CC3243	*rrnB*	ATTAATACGACTCACTATAGGGACTCACTCACTCTTTTAAAACTCACTCACT
CC3244	*rrnB*	TAGGCAGTCCTACCGGTTTTTGAATTCACTAGTAAACGCGGCAGGCAGCTCTAGAGTTAA
CC3245	*kapD*	TTAACTCTAGAGCTGCCTGCCGCGTTTACTAGTGAATTCAAAAACCGGTAGGACTGCCTA
CC3246	*kapD*	CCATAACTTTAGGGTTATCGAATTCGCATGCGACGTAGGAAACGTAGTCAGG

Table S4

Heat resistance	Percent spores surviving 80°C x 10 min
WT	87.6 +/- 10.8 %
Δ*kapD*	86.8 +/- 11.7 %

Germination rate	Time for loss of half OD600
WT	18.5 +/- 2.2 min
Δ*kapD*	19.7 +/- 2.1 min

Heat resistance assay: spores (2 OD_{600}/mL) in Tris buffer were treated at 80°C for 10 mins in a water bath, diluted to 10^{-5} and 10^{-6} in Difco DSM medium and 100 uL aliquots spread on LB plates that were incubated at 37°C for viable colony counting the following day. Germination assay: spores (2 OD_{600}/mL) were treated at 70°C for 10 mins in a water bath, incubated at RT for 15 mins and induced to germinate by addition of amino acid solution according to Nicholson and Setlow (1). The rate of decrease of OD600 was followed for 40 mins.

1. Nicholson, W.L. and Setlow, P. (1990) In Harwood, C. R. and Cutting, S. M. (eds.), *Molecular Biological Methods for Bacillus*. John Wiley and Sons, Chichester, pp. 391-450.

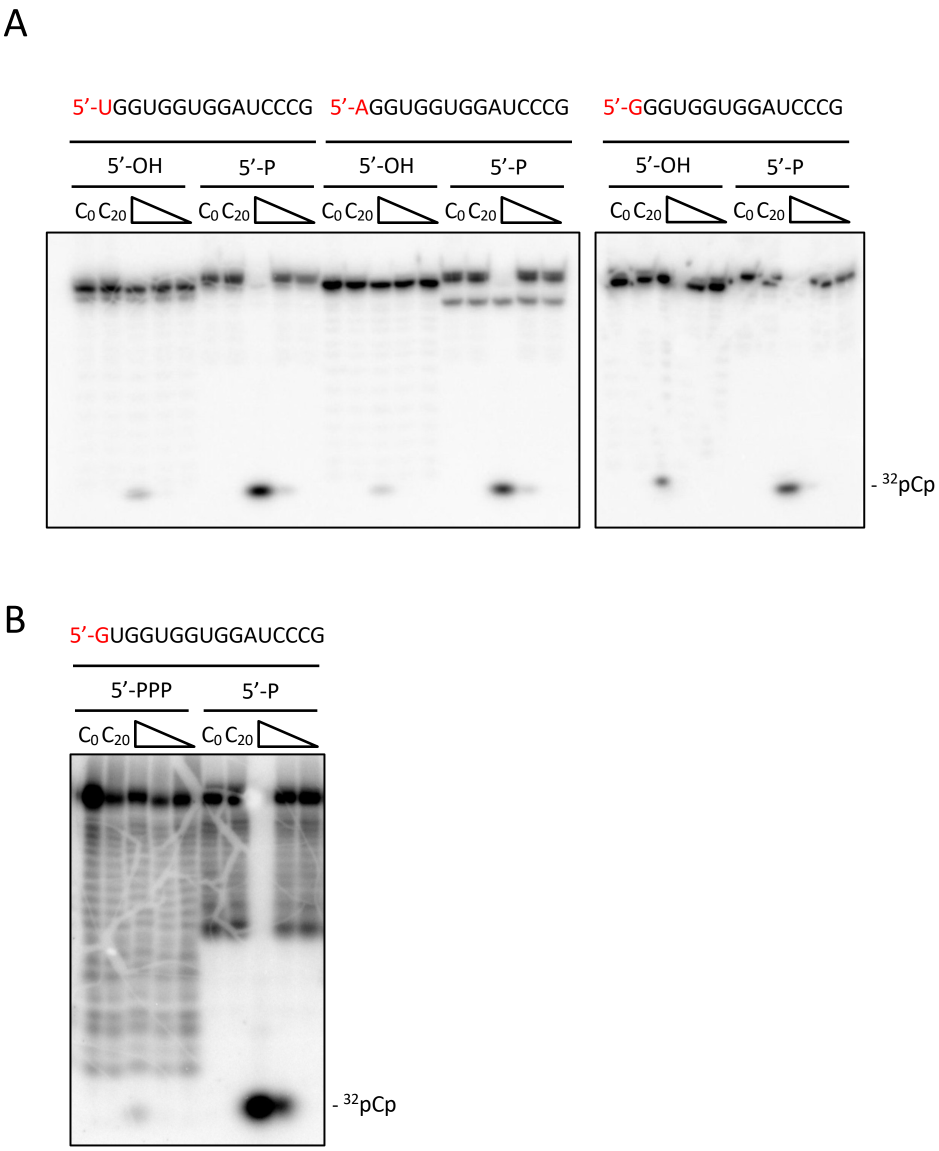

Figure S1. (A) *In vitro* activity assay performed on 3'-labelled synthetic 15-mer RNAs bearing a 5' hydroxyl (5'-OH) or a 5' monophosphate (5'-P) and with U (CCR018), A (CCR034) or G (CCR036) in the 5'-position. Reactions were performed with a 10-fold serial dilution of KapD from 10^{-1} µg/µl to 10^{-3} µg/µl final concentration, represented by right angled triangles. The migration position of ^{32}pCp is indicated. (B) KapD activity is inhibited by a 5'-triphosphate. *In vitro* activity assay performed on a 16-mer 3'-labelled synthetic RNA bearing a 5' triphosphate (5'-PPP) or a 5' monophosphate (5'-P). The RNA was *in vitro*-transcribed using a double-stranded template made by hybridizing oligos CC2524 and CC2525 together. The 5'-PPP transcript was treated with CIP and the T4 PNK to generate the 5'-P derivative. Reactions were performed with a 10-fold serial dilution of KapD from 10^{-1} µg/µl to 10^{-3} µg/µl final concentration, represented by right angled triangles. The migration position of ^{32}pCp is indicated.

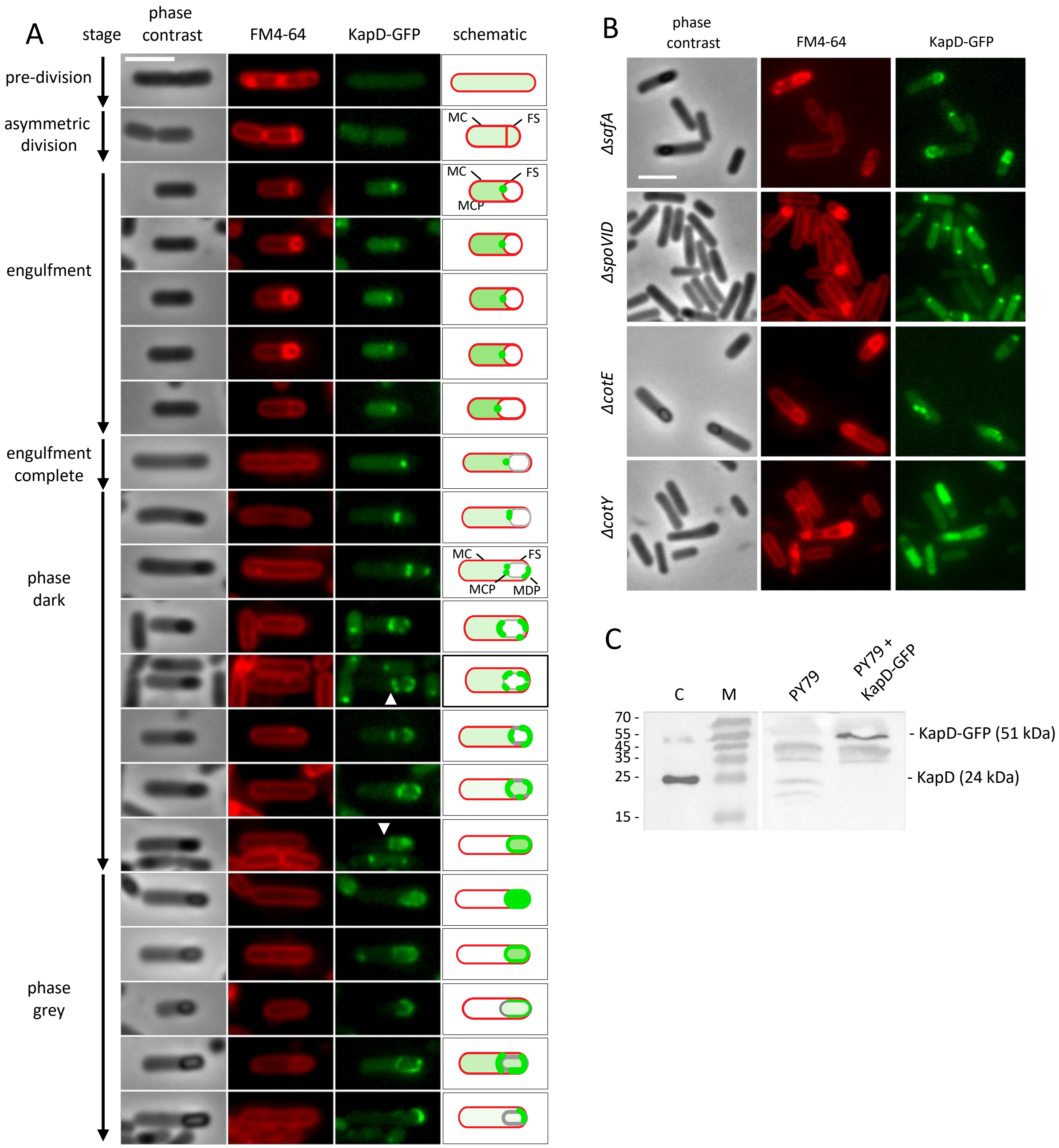

Figure S2. KapD is first expressed in the mother cell before localizing to the spore coat in a CotY-dependent manner. (A) Additional phase contrast and fluorescence microscopy images of cells expressing the KapD-GFP fusion at different morphological stages of sporulation. Membranes were stained with FM4-64 (red). A schematic representation of the cell is shown in the right panel. FS, forespore; MC, mother cell; MCP and MCD, mother cell proximal and distal poles, respectively. (B) Additional phase contrast and and fluorescence microscopy images of cells expressing a translational KapD-GFP fusion in mutants for the indicated morphogenetic proteins. All scale bars are 3 µm. In images in panels A and B where there are multiple cells, an arrowhead indicates the schematized cell shown in the right panel. (C) Western blot showing that the GFP moiety remains attached to KapD in spores expressing the KapD-GFP fusion. Lane C shows purified KapD; lane M contains molecular weight markers. The experiment was done in a *B. subtilis* PY79 background which shows an identical KapD localization profile to W168.

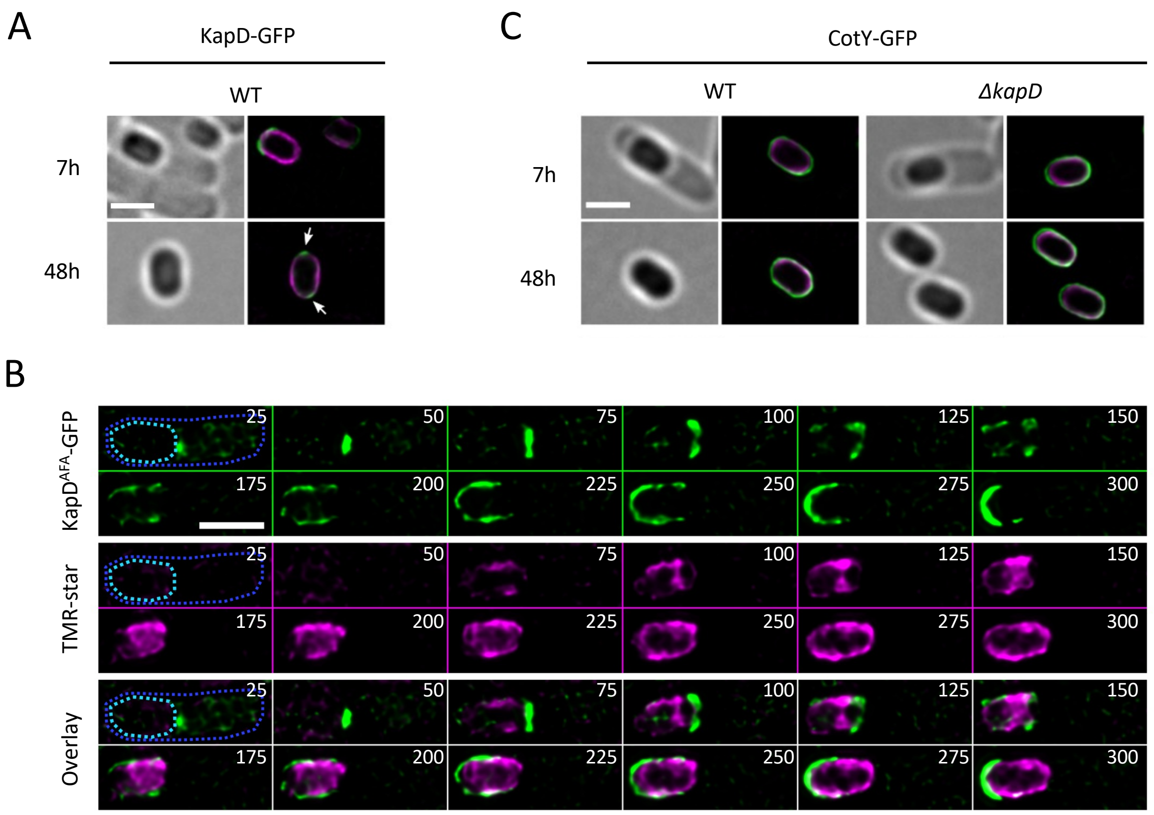

Figure S3. (A) Localization of KapD-GFP and TMR-star (pink) in WT pre-spores by SIM^2 super-resolved microscopy 7 hours (h) and 48h after induction of sporulation. Scale bar, 1 µm. (B) Single cell time-lapse showing the localization of the catalytic variant KapD^{AFA}-GFP (green) and TMR-star (pink) by SIM^2 super-resolved microscopy. Images are shown at 25 min intervals with times indicated on each panel. Scale bar, 1 µm. (C) Localization of CotY-GFP and TMR-star (pink) in WT and Δ*kapD* pre-spores by SIM^2 super-resolved microscopy 7 hours (h) and 48h after induction of sporulation. Scale bar, 1 µm. All data shown are representative of at least two independent experiments. Experiments in panels A and C were done in the PY79 strain background.

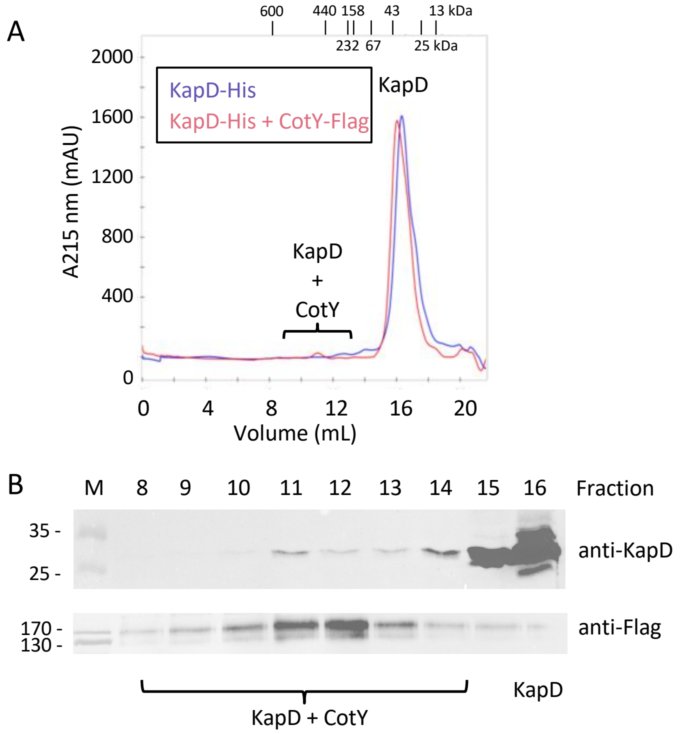

Figure S4: KapD interacts with CotY in the presence of RNase A. (A) Gel-filtration profiles of KapD alone (blue) and co-purified with CotY (red) in the presence of RNase A (10 µg/mL). The migration positions of molecular weight markers are shown above the chromatogram. (B) Western blot analysis of fractions 8-16 from the gel-filtration column of KapD + CotY probed with an anti-KapD antibody (upper panel) and an anti-Flag antibody (lower panel). The position of molecular weight standards is shown on the left side of each panel.

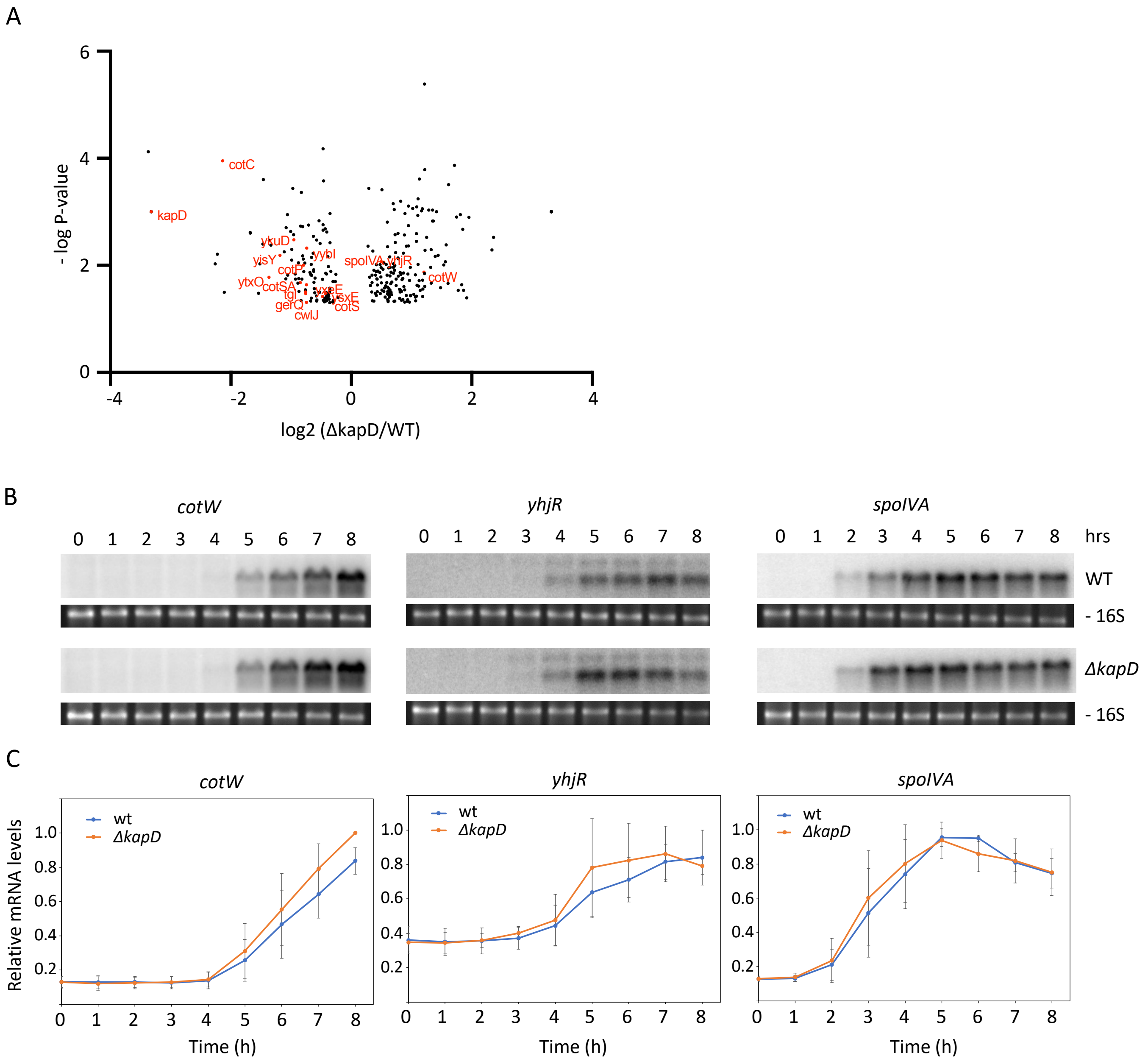

Figure S5. Changes in spore coat composition are not directly attributable to KapD RNase activity. (A) Volcano plot of data from Table S1 showing major changes in spore coat composition in strains lacking KapD. Known spore coat proteins are labelled in red. Only candidates with a P-value >0.05 are shown. Candidates that were not detected in either WT or Δ*kapD* strains were arbitrarily attributed a fold-change of 10 and a P-value of 0.001. (B) and (C) Expression of *cotW*, *yhjR* and *spoIVA* mRNAs are similar in WT and Δ*kapD* strains over the sporulation cycle. (B) Northern blot showing expression of *cotW*, *yhjR* and *spoIVA* in total RNA isolated from cells 0-8 hrs after resuspension in sporulation medium, probed with oligos CC2674, CC1837, CC2681, respectively. Ethidium bromide stained 16S rRNA is shown under each gel as a loading control. Note that the *cotW* and *yhjR* membranes have the same loading control. (C) Quantification of three independent experiments such exemplifed by that shown in panel A. Signals were normalised to the maximal value for each membrane containing both WT and Δ*kapD* samples. Error bars represent standard deviation.

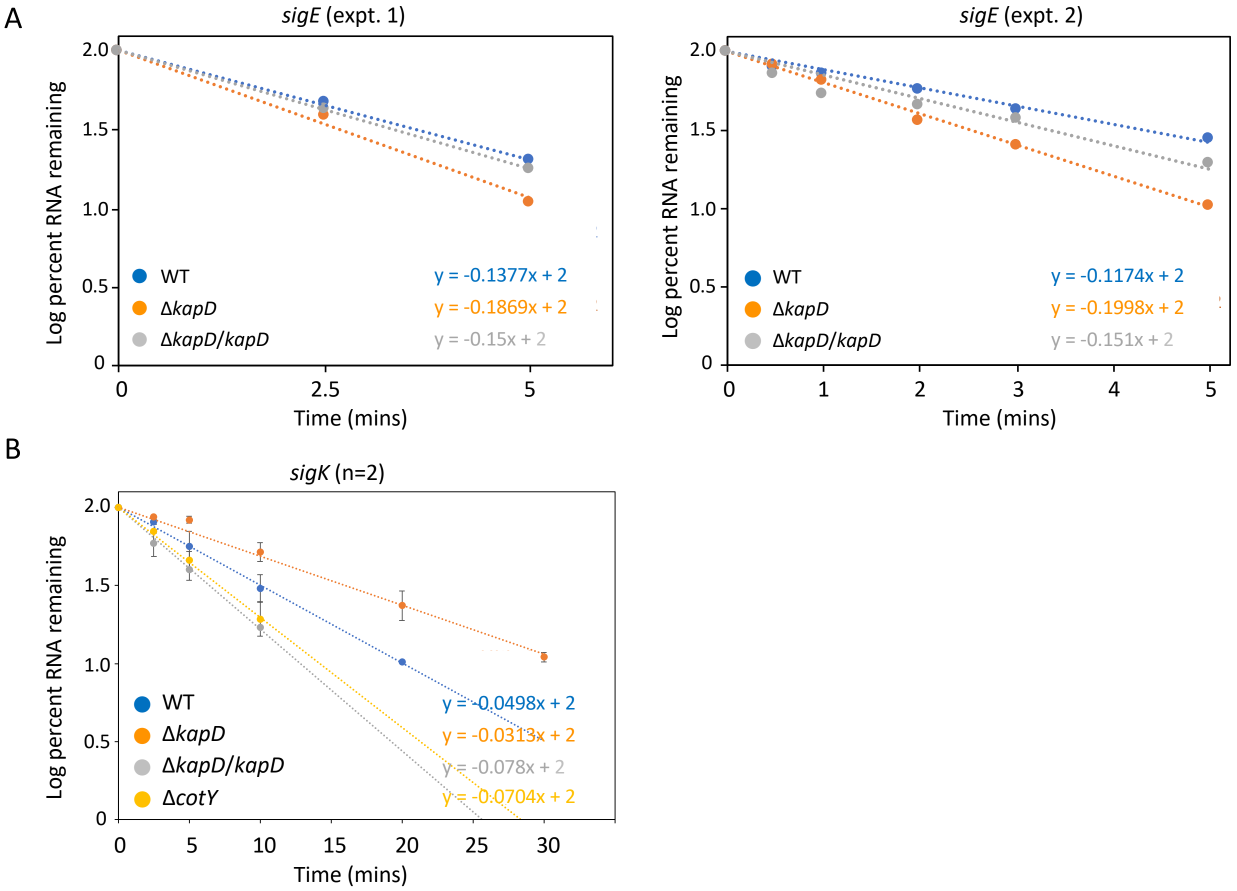

Figure S6. The stability of the *sigK* mRNA is KapD-dependent (A) Quantification of two independent experiments (expt.) measuring levels of (A) *sigE* and (B) *sigK* mRNAs remaining at times after rifampicin addition to cells four (T4) and five (T5) hours, respectively, after resuspension in sporulation medium.

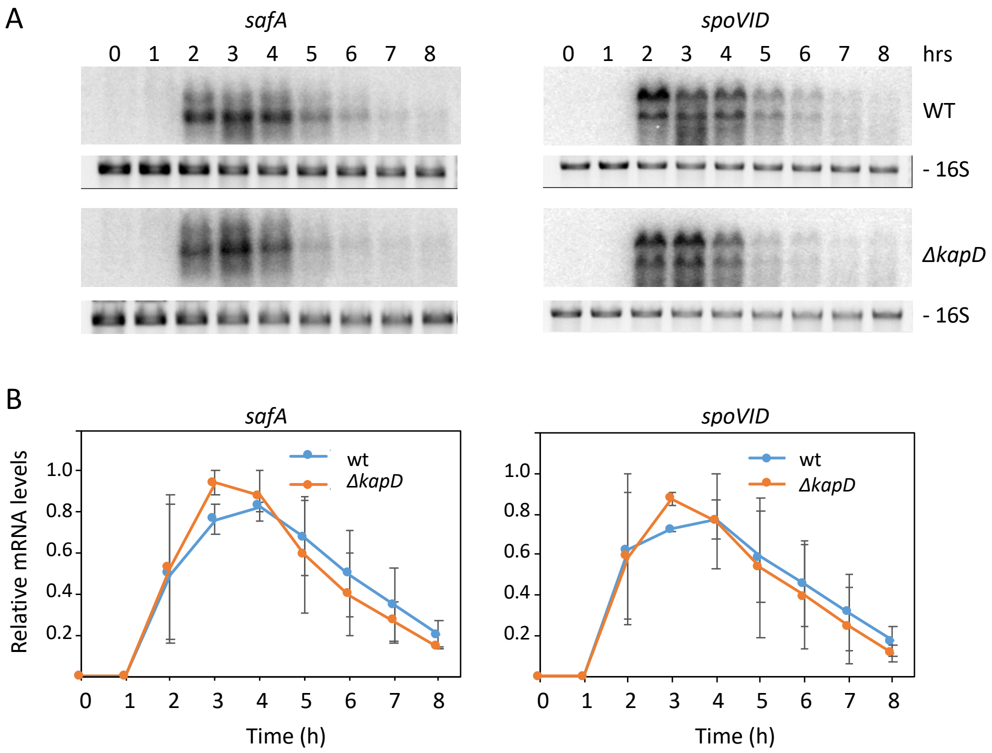

Figure S7. Expression of *safA* and *spoVID* are similar in WT and Δ*kapD* strains over the sporulation cycle. (A) Northern blot showing expression of *safA* and *spoVID* in total RNA isolated from cells 0-8 hrs after resuspension in sporulation medium, probed with oligos CC2682 and CC2689, respectively. Membranes were stripped and reprobed for16S rRNA (oligo CC058) as a loading control. (B) Quantification of two independent experiments exemplifed by that shown in panel A. Signals were normalised to the maximal value for each membrane containing both WT and Δ*kapD* samples. Error bars represent standard deviation.
